# Endoplasmic Reticulum Redoxome: Protein Folding and Beyond

**DOI:** 10.1021/acs.biochem.5c00527

**Published:** 2025-12-12

**Authors:** Percillia V. S. Oliveira, Tiphany C. De Bessa, Francisco R. M. Laurindo

**Affiliations:** Laboratorio de Biologia Vascular (LVascBio), LIM-64, Instituto do Coracao (InCor), Hospital das Clinicas HCFMUSP, Faculdade de Medicina, 42523 Universidade de Sao Paulo, Sao Paulo, São Paulo CEP 05403-000, Brazil

**Keywords:** protein disulfide isomerase, NOX, NADPH oxidase, vascular smooth muscle cells, phenotype, oxidants

## Abstract

The endoplasmic reticulum (ER), the largest cellular organelle, is crucially dependent on its redox organization. First, to optimize disulfide bond formation in nascent proteins, it maintains a relatively oxidizing environment, reminiscent of the extracellular space. Second, it harbors several oxidoreductases from the protein disulfide isomerase (PDI) family, together with Ero1α oxidase and chaperones, which compose interplaying oxidative, reductive, and chaperone pathways to optimize protein processing. Third, disulfide formation and reshuffling in client proteins, involving thiol oxidation and disulfide exchange reactions, connect proteostasis to ER/cellular redox homeostasis. ER redox folding involves Ca^2+^-dependent liquid phase separation of PDI complexes. Calcium fluxes heavily interplay with dynamic redox regulation. ER stress disrupts the ER redox state and, in turn, is also regulated by cellular redox processes. Moreover, the ER makes membrane contacts with many other organelles such as plasma membrane, peroxisomes, and mitochondria, which are hubs for mutually dependent oxidant and calcium-linked effects. Furthermore, the ER redoxome extends to other subcellular and extracellular locations, a process we termed the “ER-dependent outreach redoxome (ERDOR)”. ERDOR can occur by overflow of ER products such as H_2_O_2_, mobility of ER-associated domains or, mainly, via ER oxidoreductase translocation. The ER establishes a particular communication with the extracellular milieu via translocation of PDIs. Despite the low levels of extracellularly located ER oxidoreductases, they redox-regulate several molecular targets and may compose a peri/epicellular redox network. This article provides a comprehensive overview of the ER redoxome as an important emerging frontier to understand not only redox proteostasis but also intra- and intercellular redox communication.

## Graphical abstract

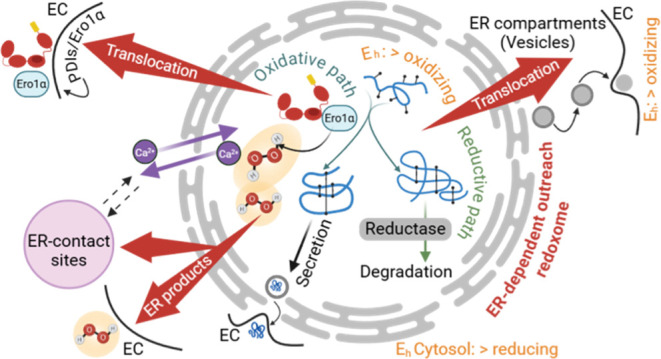

The endoplasmic reticulum (ER) is the largest cellular organelle, with functions including protein folding and processing, lipid metabolism, calcium regulation, interorganelle integration, endomembrane redistribution, and response to pathogens.^1^ Most of its functions merge with redox pathways, making the ER an increasingly evident player in cellular redox (patho)­physiology. The ER redox landscape converges with its optimization for protein folding.^2^ ER redox pathways also interplay with those of most other organelles and subcellular compartments outside of the ER.^3^ Specifically, redox cross-talk between ER and extracellular (EC) milieu is crucial for intercellular redox communication.^4^ This review addresses an overview of such an ER redoxome scenario. We emphasize discussions of the disulfide and protein-folding-related landscapes, major determinants of the ER redoxome. We then move to discuss ER-organelle contacts as integrative hubs for redox communication and end up with a discussion of the ER-dependent outreach redoxome.

## Oxidative Protein Folding: Disulfide Bond Formation

Secretory or membrane proteins contain disulfide bonds within and between forming polypeptide chains, a crucial step for their correct folding and stability, in a process termed oxidative folding.^5^ Disulfide bonds insertion into nascent polypeptides occurs mainly in the ER lumen of eukaryotes and periplasmic space of prokaryotes.^5–7^ The ER folds and processes proteins destined to travel or those residing in the secretory pathway, ca. one-third of all proteins.^8^ As such, the ER is endowed with a quality and quantity control network orchestrating protein synthesis, translocation, folding, assembly, post-translational modifications, sorting, and degradation. Of note, in specialized secretory cells—goblet cells,^9^ pancreatic β cells,^10^ and plasma cells^11^—the total daily protein output from the ER can surpass their entire cellular mass.

Disulfide bonds are a highly frequent covalent link in proteins, second only to peptide bonds.^12,13^ Disulfides are evolutionary conserved along evolution, with minimal loss over time.^12,14^ Disulfide bond formation is based on redox reactions in which two cysteine (Cys) residues are oxidized by two-electron removal, yielding a covalent bond (rev. in refs 5 and 7 ). Most disulfide bonds form during protein folding through enzyme-dependent thiol–disulfide exchange reactions in which an oxidized thiol oxidoreductase donor transfers the bond to a reduced protein substrate acceptor via the formation of a transient mixed disulfide intermediate (Figure 1b). Disulfides are ubiquitously distributed among proteins, being highly abundant in proteins harboring an N-terminal secretion signal sequence from the ER/secretory system, EC milieu, and mitochondrial intermembrane space.^14^ Some disulfides may potentially accumulate at ER-derived compartments and eventually in membrane-less organelles. Disulfides are also found in other cell compartments, including nucleus and cytosol; the latter includes, e.g., superoxide dismutase (SOD1)^14^ and Keap1.^15^ Contrary to the former belief that disulfides were inert, mounting evidence indicates regulatory roles beyond their basic structural function of stabilizing protein architecture.^13,16,17^ Indeed, cleavable disulfide bonds termed allosteric disulfides (rev, by refs 14 and 17 ) reversibly affect the function of mature proteins in which they reside, while not being directly catalytic. Allosteric disulfide formation/cleavage governs protein conformational changes implicated in robust functional effects.^14,16,18^

**Figure 1:**
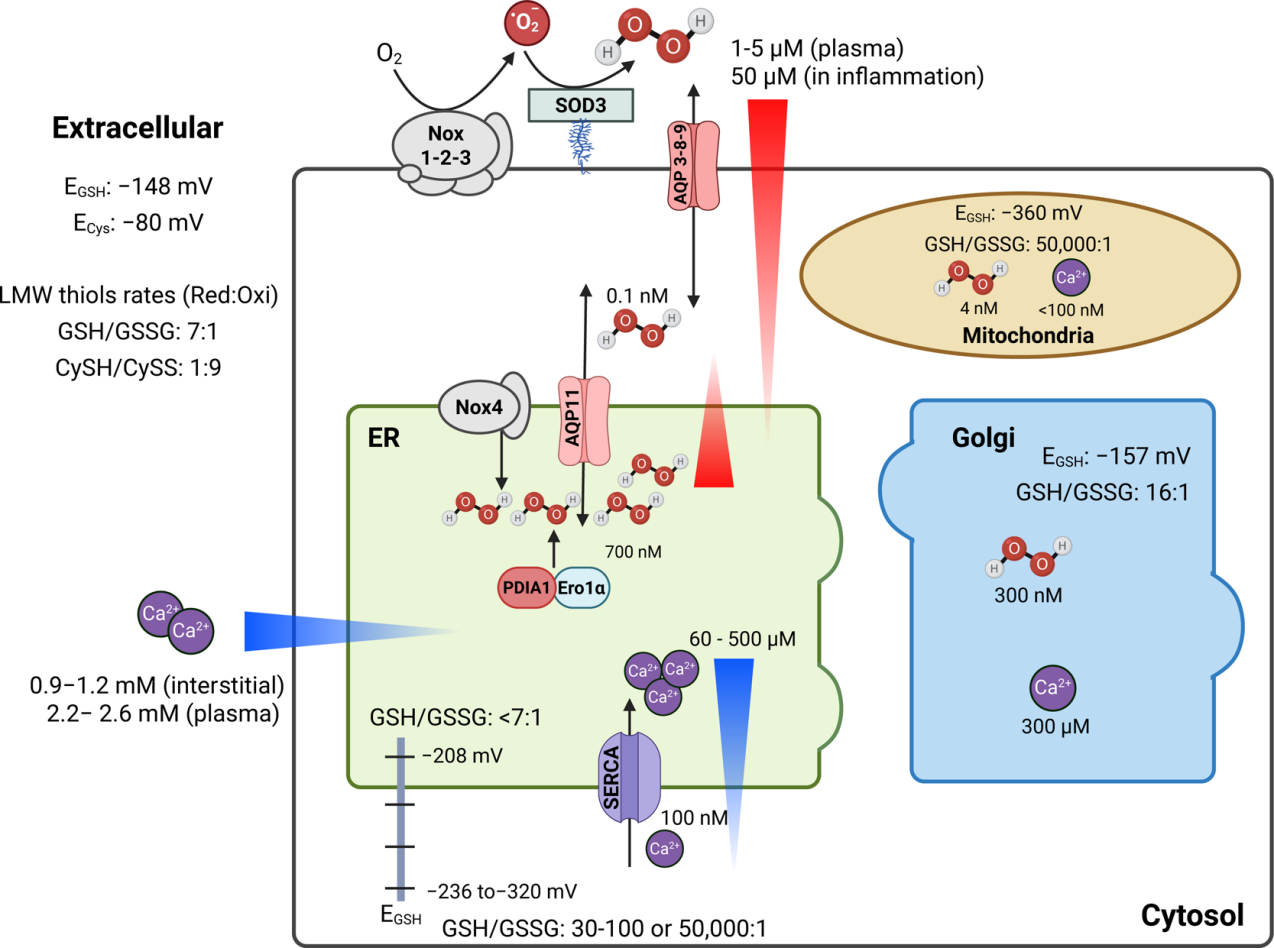
Schematic overview of the redox poise and calcium levels of intra- and extracellular (EC) compartments. In line with their evolutionary connection, there are relative similarities between the ER and EC redox milieu. Both are substantially more oxidizing than the cytosol, while the EC redox potential is even more oxidizing. The major intracellular thiol–disulfide redox buffer is glutathione with its reduced (GSH) and oxidized (GSSG) forms. Total glutathione levels in the cytosol range between 10 and 30 mM, while reported levels in the ER are ca. 15 mM.^26,27^ Their corresponding redox potentials (*E*_GSH_) yield −320 mV and −208 to −236 mV, respectively.^26–31^ The cytosolic GSH/GSSG ratio is strongly reducing, with reported values of 30:1, 100:1,^32^ and more recently 50,000:1. The cytosolic GSSG concentration was reported as ca. 200 nM,^33^ in line with prior evidence for nanolow μM levels. In contrast, the ER GSH/GSSG ratio has been reported to be < 7:1, in accordance with its more oxidizing environment.^26^ GSH and GSSG may be transported into the ER by facilitated diffusion through the Sec61 translocon in a Kar2 (yeast Bip/Grp78) redox-dependent fashion.^27^ It remains an open question whether mammalian cells employ an analogous mechanism. Additionally, GSH transport into the ER may also occur through the ryanodine receptor channel (RyR) 1.^34,35^ There is currently no model to explain the potential functional interplay between GSH transport via RyR1 and Sec61 translocon. The ER is the major reservoir of GSSG in cells; however, it is unclear whether GSSG is specifically exported from the ER to the cytosol. The ER-localized acetyl-CoA transporter SLC33A1 ^36^ modulates GSSG pool in Keap1 mutant cells.^37^ Mitochondria, similar to the cytosol, have a more reducing redox potential, while the Golgi complex is even more oxidizing than the ER. Key systems for H_2_O_2_ generation are shown for the ER (Nox4, PDIA1-Ero1 axis) and EC milieu (Nox 1,2,3-dependent superoxide radical (O_2_^•–^), coupled to superoxide dismutation by SOD3 into H_2_O_2_). Levels of H_2_O_2_ at various subcellular sites and EC milieu are shown, including their transporting channels (peroxiporins and aquaporin (AQP)). In parallel, ER calcium levels are quite high compared to other compartments such as Golgi and mitochondria. Importantly, these data significantly depend on the cell type, physiological conditions, and methods used and may vary to a good extent. All values summarized here are from the following references.^33,38–43^ Created in BioRender.

## ER: A Special Organelle Optimized for Protein Folding

The ER consists of an interconnected membrane network forming tubular and cisternal structures enclosing a single continuous lumen.^1^ The ER lumen, topologically equivalent to the EC milieu, may have evolved through plasma membrane invagination.^19^ It is referred to as “the EC space within the cell”, given its high calcium concentration and a more oxidizing redox state relative to the cytosol and other organelles, expressed by their opposite patterns of glutathione levels and redox potentials,^20–22^ detailed in Figure 1. Such an ER oxidizing shift is thought to facilitate the oxidative folding and processing of complex proteins.

The ER lumen has several highly concentrated proteins (∼100 mg/mL)^23^ acting as folding factors, namely, molecular chaperones (e.g., Grp78, ∼0.5 mM^24^) and thiol–disulfide oxidoreductases (e.g., PDIA1, ∼0.2–0.5 mM^3^), crucial for folding and disulfide insertion, respectively, in nascent proteins.^19^ In Hela cells, when normalized to Grp78, relative abundances of other molecular chaperones such as Grp94 and calnexin (CNX) are ca.91 and 57, respectively, while the thiol-oxidoreductases ERp57 (ca.70) and ERp5 (ca.21) are comparatively less abundant.^25^ In mammalian cells, proteins are driven to the ER via an N-terminal signal sequence targeting them to the translocon channel (Sec61) in the ER membrane. The signal peptide is cleaved from the nascent polypeptide as it emerges from this channel into the ER lumen and undergoes subsequent folding, processing, maturation, and quality control. These processes are supported by precise adjustments in the redox potential and calcium. Upon achieving their final conformation (*native state*), they are sorted for secretion or exposed at the cell surface.^20,22^

### Folding Factors: ER Resident Chaperones and Thiol Oxidoreductases

The ER harbors analogs of major chaperones found in cytosol and mitochondria, such as Hsp40, Hsp70, Hsp90, and Hsp100, as well as the CNX/calreticulin (CRT) families. Others—such as Hsp60 and small heat shock proteins (sHSPs)—are absent in the ER lumen, with some exceptions in plants.^44^ The most abundant chaperones include BiP/Grp78 and Grp94, which are homologous to cytosolic Hsp70 and Hsp90, respectively. Grp78 facilitates protein folding via its binding to short hydrophobic patches exposed in folding intermediates, misfolded proteins, or unassembled oligomer subunits. As an ATPase, its chaperone activity requires ATP/ADP exchange linked to conformational changes critical for substrate folding.^45,46^ Although the ER does not synthesize ATP itself, it relies on ATP produced primarily in mitochondria and cytosol. The precise import mechanisms remain yet unclear, while several potential routes have been proposed.^47^ Recent evidence^48^ reinforces robust support for a role of the ER transmembrane protein SLC35B1 as an ATP transporter. Similar to other Hsp70s, Grp78 activity is regulated by J-domain proteins from the Hsp40 family, which includes several ER-resident members.^49^ Under oxidative conditions, oxidation of Grp78 conserved Cys residue by both H_2_O_2_ and GSSG enhances its holdase activity, preventing protein aggregation and supporting cell survival.^50,51^ Meanwhile, Grp94 exists as a constitutive dimer stabilized by its C-terminal domain.^52,53^ Its ATP-dependent chaperone activity, specially focused on the late stages of protein folding,^54^ targets a more selective number of clients than other ER chaperones, including insulin-like growth factors I and II.^54,55^

Thiol-oxidoreductases are a major group of folding factors unique to the ER, responsible for the formation and reshuffling of disulfide bonds during folding mediated by members of the protein disulfide isomerase (PDI) family. PDIs bear functional equivalents in the bacterial periplasm, the Dsb family proteins;^56,57^ however, many PDIs have evolved to consolidate thiol oxidase, reductase, and isomerase activities within the same protein, while these functions are mainly separated among distinct Dsbs.^57,58^ The PDI family currently comprises 24 proteins (Table 1) that differ in size, domain arrangement, expression, subdomain localization (i.e., ER lumen, ER membrane, cis-Golgi), and function. While all family members presumably associate with thiol–disulfide exchange, based on the well-known roles of the prototypical founding member PDIA1, not all are true orthologs able to fully perform these reactions. Such are the evolutionarily related paralogs (such as AGR2, ERp44, TMX2) possessing the thioredoxin (Trx)-like fold structure but not the canonical dithiol redox motif (CXXC), which is the catalytic motif that reacts with thiols or disulfides in client proteins.^59,60^ The Trx fold, a unifying signature of all PDIs, is a structural motif (βαβαβαββα, i.e., a central five-stranded β-sheet surrounded by four α-helices) shared by all Trx superfamily members. PDI family members contain variable combinations of catalytically active (a-type) domains harboring CXXC-like motifs and catalytically inactive (b-type) domains. The prototypic a-type motif is Cys-Gly-His-Cys (CGHC), but distinct PDIs show many variations among the intervening amino acids (Table 1), likely affecting the reactivity of associated Cys and substrate specificity.^3,4^ In the extreme cases of ERp44 and TMX2, e.g., the redox-active motifs lack, respectively, C- or N-terminal Cys residues. However, the single Cys in ERp44 ^61^ can form mixed disulfides with clients, while TMX2 targets are unclear.^62^ In contrast, ERp27 and ERp29 do not contain redox-active motifs at all (Table 1). Meanwhile b-type domains have no redox Cys and are usually enriched in hydrophobic residues involved in substrate recognition and binding, especially the b′ domain hydrophobic pocket.^3,59,60^ In addition to intrinsic PDI architecture, their substrate specificity may be regulated via interactions with chaperones—such as ERp57 with the CNX/CRT cycle^63^ and ERp5 with Grp78.^64^

**Table 1:** An Overview of PDI Family Members[Table-fn t1fn9]

name	other names	retrieval sequence	length	domains	redox-active motifs	N-glycosylation	Asp/Glu-rich Ca^2+^-binding domain	linker region	transmembrane domain
PDIA1	PDI, P4HB	AV-KDEL	508 aa	a-b-b′-a′	CGHC (a, a′)	no^a^	Yes	yes	no
PDIA2	PDIp, PDIA2, PDIR	GS-KEEL	525 aa	a-b-b′-a′	CGHC (a), CTHC (a′)	yes	No	yes	no
PDIA3	ERp57, GRP58	KA-QEDL	505 aa	a-b-b′-a′	CGHC (a, a′)	no	No	yes	no
PDIA4	ERp72	RT-KEEL	645 aa	a^0^-a-b-b′-a′	CGHC (a^0^, a, a′)	no	Yes	yes	no
PDIA5	PDIr	KK-KEEL	519 aa	b-a-a-a	CSMC, CGHC, CPHC	no	No	no	no
PDIA6	ERp5, P5, TXNDC7	LG-KDEL	440 aa	a-a^0^-b	CGHC (a, a^0^)	no	Yes	no	no
PDIA7	PDILT	KV-KEEL	584 aa	a-b-b′-a′	SKQS (a), SKKC (a′)	yes	No	yes	no
PDIA8	ERP27, ERp27	TP-KVEL	273 aa	b–b′	none	no^b^	No	no	no
PDIA9	ERP29, ERp28	AE-KEEL	261 aa	a	none	no^b^	No	no	no
PDIA10	ERP44, TXNDC4, ERp44	RD-RDEL	406 aa	a-b-b′	CRFS	no^c^	No	no	no
PDIA11	TMX1, TXNDC1	(AI-RQR-XX)^g^	280 aa	a-like	CPAC	no	No	no	yes
PDIA12	TMX2, TXNDC14	(EN-KKDK)^h^	296 aa	a-like	SNDC	no^b^	No	no	yes
PDIA13	TMX3, TXNDC10	(LE-KKKD)^h^	454 aa	a-like	CGHC	yes	No	no	yes
PDIA14	TMX4, TXNDC13	(SL-RQR-XX)^g^	349 aa	a-like	CPSC	yes	Yes	no	yes
-	TMX5, TXNDC15	none	360 aa	a-like	CRFS	yes	No	no	yes
PDIA15	ERp46, TXNDC5	QA-KDEL	432 aa	a^0^-a-a′	CGHC (a^0^, a, a′)	no	No	no	no
PDIA16	ERp19, ERp18, AGR1, TXNDC12	HL-EDEL	172 aa	a-like	CGAC	no^d^	no	no	no
PDIA17	AGR2, HAG-2	LL-KTEL	175 aa	a-like	CPHS	no^e^	No	no	no
PDIA18	AGR3, HAG-3	LI-QSEL	166 aa	a-like	CQYS	no^d^	No	no	no
PDIA19	ERdj5, DNAJC10	RN-KDEL	793 aa	Trx1–Trxb1–Trxb2–Trx2–Trx3–Trx4	CSHC (Trx1), CPPC (Trx2), CHPC (Trx3), CGPC (Trx4)	yes^d^	Yes	no	no
PDIB1	CASQ1, calsequestrin 1	none	396 aa	b-b-b′	none	yes	Yes	no	no
PDIB2	CASQ2, calsequestrin 2	none	399 aa	b-b-b′	none	yes	Yes	no	no
ERp90	TXNDC16	RR-DKEL-XX	825 aa	Trx1–Trx2–b–Trx3-Trx4–Trx5–b	CX_8_C (Trx1), CX_9_C (Trx2), CX_6_C (Trx3)	yes	No	no	no
TXNDC11	EFP1	none	985 aa	Trx1–Trx2–Trx3–Trx4–Trx5	CGQS (Trx1), CGFC (Trx5)	yes	no^f^	no	yes

a Gene name is underlined. We included TXNDC16 that does not have the canonical CXXC active site motif contains other conserved Cys which could endow the protein with redox activity.^173^

b O-glycosylation reported in T cells;^339^ N-glycosylation reported (Glyco-PDIA1-artificially inserted N-glycan site).^69^

c Canonical N-glyco site.

d O-glycosylation reported in HeLa cells.^340^

e Reported site for O-glycosylation.

f O-glycosylation reported in AGR2 secreted by mammary epithelial cells.^341^

g Coiled-coil region at the C-terminus.^342^

h Diarginine motif RXR type for ER retention.

I KKXX-type sequence for ER retrieval of type I transmembrane proteins XX-any amino acid.

While most clients leave the ER after proper folding, folding factors are mostly retained within the ER, mainly via two mechanisms: (a) their association with multiple other ER-resident proteins and (b) the presence of a C-terminal tetrapeptide ER-retrieval signal. The latter is another distinctive PDI family feature, consisting of a Lys-Asp-Glu-Leu (KDEL) motif or variants thereof—RTEL, KDEL, QEDL,^65,66^ while 3 members do not contain any ER retention motif: TMX5, CASQ1, and CASQ2 (Table 1). As KDEL-bearing proteins escape the ER, they bind specific receptors (KDEL receptors, KDELRs, comprising KDELR 1, 2 and 3) in the cis-Golgi.^67,68^ KDELRs continuously cycle within the early secretory pathway (that is, the ER, cis-Golgi, and ER-Golgi intermediate compartment, ERGIC), first capturing KDEL-chaperones that arrive at the ERGIC/Golgi and then retrieving and releasing them back into the ER.^61^ How nonabundantly expressed KDELRs can prevent the secretion of highly abundant ER proteins is intriguing. An interesting model conceptualizes the early secretory pathway as a chromatographic column,^61,66^ where selective sorting and partitioning of cargo molecules occur based on their tendency to interact with the ER matrix, forming supramolecular complexes with different diffusibility. As a major folding factor, PDIA1-containing complexes would be upstream in the column and more static, while quality control components like ERp44 would be distal, cycling fast between ER and Golgi.^61,66,69^ However, KDEL motif deletion in PDIA1 limits its secretion, the corresponding ERp44 mutant is rapidly secreted,^69,70^ reflecting both its weaker affinity for the ER matrix and its engagement with forward cargo receptors.^61,66,71^ Despite these mechanisms avoiding ER protein transport beyond the Golgi, certain KDEL-bearing proteins including PDIA1 are actively secreted by living cells^69,72^ with important (patho)­physiological implications.^12^ While ER oxidoreductases have no analogs in cytosol and mitochondria, such mobility within and outside cells imparts them important roles in the regulation of the ER-dependent outreach redoxome, discussed in the next sections. While the PDI family and related partners are the main ER thiol-oxidoreductases, recent work highlighted the non-PDI family cysteine-rich epidermal growth factor-like domain (CRELD) protein family CRELD2. This ubiquitous ER-resident protein has many CXXC motifs able to perform thiol–disulfide exchange reactions akin to a PDI-like activity^73,74^ and forms disulfide-linked complexes with other PDIs.^74^ CRELD2 displays a C-terminal KDEL-like motif (REDL) and is robustly secreted extracellularly via Golgi pathways upon ER stress.^73,75,76^ Associated physiological effects include protection against ER stress and angiogenesis after myocardial infarction.^77^

In addition to disulfide insertion, N-glycosylation is another key component of ER-mediated protein processing, estimated to occur in >7000 proteins.^78^ While glycosylation occurs in many cell compartments, cotranslational attachment of N-glycans is ER-specific, with many chaperones dedicated to folding/quality control of nascent glycoproteins. CNX and CRT are the most well-studied ER-exclusive lectin chaperones, recruited by N-glycans to nascent proteins. They are homologous, with similar structure and function, although-CNX is membrane-anchored and CRT a luminal protein.^6,79^ CNX and CRT ensure proper glycoprotein folding via their binding to oligosaccharide-processing intermediates having a single terminal glucose residue (monoglucosylated N-glycans).^6,23^ These proteins also bind ATP, Ca^2+^, Zn^2+^, and the PDI family member ERp57.^80–82^

### Dynamic Calcium Fluxes

The ER maintains luminal Ca^2+^ concentrations ranging from 100 to 500 μM, ca. 1000-fold greater than cytosolic levels (∼100 nM),^83,84^ in line with its role as the primary dynamic Ca^2+^ storage compartment (Figure 1). Together with mitochondria, the ER forms a coordinated system for generating regulated Ca^2+^ signals for many cellular processes.^83,85^ Ca^2+^ release from the ER relies on the gated channels RyR and inositol 1,4,5-trisphosphate receptor (IP3R), while ER Ca^2+^ uptake from cytosol depends on sarco/ ER Ca^2+^ ATPase (SERCA) pumps.^86^ Other pathways affecting ER lumen Ca^2+^ levels include ER-to-cytosol Ca^2+^ leakage via several mechanisms^85,86^ and ER Ca^2+^ replenishment by influx of EC Ca^2+^ via store-operated Ca^2+^ entry (SOCE). As an ensemble, the ER-resident stromal interaction molecule (STIM) Ca^2+^ sensor proteins and the plasma membrane-associated store-operated Orai Ca^2+^ channels cooperate for SOCE.^87^ ER calcium has several implications in protein folding: ER chaperones such as CNX, CRT, Grp78, and Grp94 bind proteins in a Ca^2+^-dependent manner.^20,85^ Moreover, chaperones and thiol oxidoreductases can affect the ER-luminal Ca^2+^ content. During the unfolded protein response (UPR, see below), Grp78, which normally seals the translocon, detaches from it, leading to ER Ca^2+^ leak.^88,89^ Also, SOCE can be inhibited by STIM1 redox regulation via ERp57.^90^ These connections highlight the interplay among ER luminal redox processes, ER-resident proteins, and ER Ca^2+^ handling machinery.

Cellular redox signaling and ER Ca^2+^ levels are closely linked, in line with the general parallel between the levels of reactive oxygen species (ROS) and Ca^2+^ in distinct cell compartments (Figure 1). ER-regulated changes in the intracellular Ca^2+^ concentration support ROS production, and vice versa. This can occur via Nox NADPH oxidases—via direct Ca^2+^ binding to Nox5 and Duox1-2 ^91^—or by indirect activation of Nox1, 2, or 4.^92–94^ Interestingly, the Nox NADPH oxidase family phylogenetically evolved from common ancestors regulated by calcium.^95^ Mitochondrial Ca^2+^ uptake from the ER enhances respiratory chain activity and can affect ROS production.^96^ ROS, in turn, can modify the Ca^2+^ channels and transporters. Nox-derived hydrogen peroxide is a second messenger to activate Ca^2+^ channel receptors such as RyRs and IP3Rs.^96,97^ Further discussion of the role of ER contact sites in Ca^2+^ dynamics is provided below.

### Disulfide Bond Formation: Connecting Protein Folding and Redox Homeostasis

As discussed above, the oxidative ER environment facilitates the formation of disulfide bonds during protein folding. ER clients require the insertion of intra- or intermolecular disulfides for protein stability, especially relevant for secretory proteins after exiting the ER, as well as oligomer assembly and stabilization of intermediate structures crucial for efficient protein folding.^6,98^ Thus, correct disulfide bond formation is crucial for attaining native functional structures and avoiding protein misfolding and malfunction. This is achieved primarily through an oxidative pathway, but disulfide cleavage and rearrangements (i.e., reduction/isomerization) are also required for targeting folded proteins for degradation or correcting non-native disulfides, respectively.^83,99,100^ Thus, both oxidative and reductive pathways must be tightly regulated for efficient ER proteostasis.^101^

#### Oxidative Pathways

Although disulfide bonds can be formed in vitro, in the ER oxidative folding is catalyzed by PDI family members. PDIA1 is structured as four Trx-like domains named in sequence a-b-b′-a′, which assume a twisted U-shape. The two a-type domains (a, a′) contain the classic redox catalytic motif (CGHC) while the two b-type domains (b, b′) have no catalytic Cys. The two b′ domain Cys residues (Cys312, Cys343) cannot catalyze thiol–disulfide exchange, but may suffer redox modifications, consistent with possible physiological importance.^102^ The b-type domains are the major substrate-binding sites, with the hydrophobic binding pocket embedded into the b′ domain (Figure 2a). The hydrophobic phenylalanine-enriched β-hairpin loop in Ero1α (endoplasmic reticulum oxidoreductin 1) binds to aromatic PDIA1 residues at the b′ pocket, a mechanism that accounts for Ero1α preference for PDIA1 over other PDIs, a feature favoring its oxidation of PDIA1 a′-domain.^3,103^ In addition, a short unstructured segment of 19 residues, named X-linker, links b′ and a′ domains. The latter, in turn, connects to the highly acidic C-terminal tail ending with the ER retrieval sequence KDEL.^3^ In addition to oxidative and reductive activities, some PDIs display chaperone activity, able to prevent substrate aggregation and misfolding^3^ and enabling the folding of denatured proteins that do not contain disulfides.^104,105^ Chaperone effects are not directly linked to the catalytic CXXC groups and associate mainly with b-type domains, although a chaperone-like role for the a domain has been reported.^106^ Such chaperone effects are a basic step of PDI-assisted folding as the likely way that PDI approaches the misfolded substrates to later exert thiol redox activities.^3^

**Figure 2:**
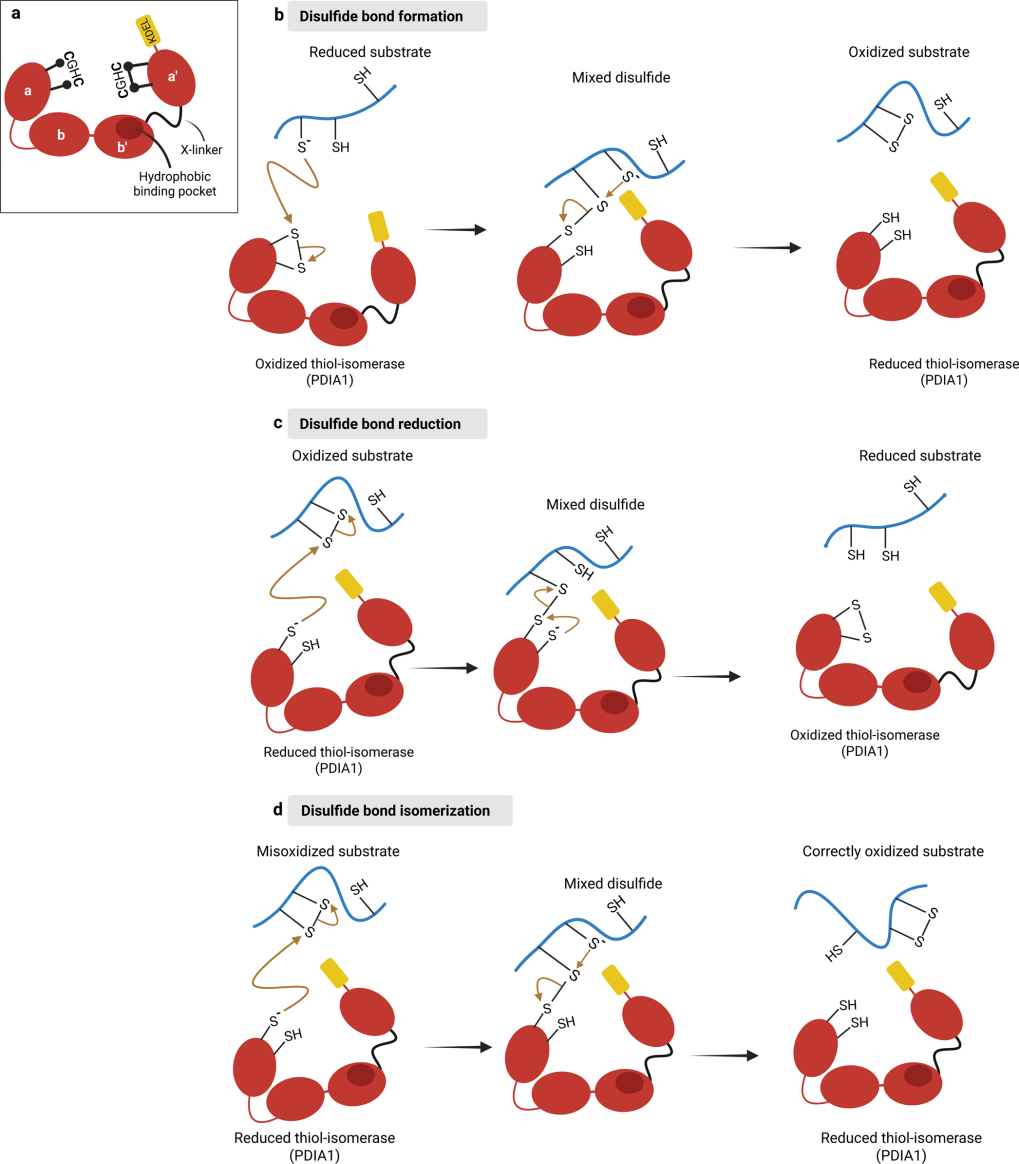
Mechanisms of PDI-mediated thiol–disulfide exchange reactions. (a) Prototypic PDI family members display tandem a and b-type domains, each containing a thioredoxin (Trx)-fold. Their redox functions within the ER are best understood through founding family member PDIA1, shown here. PDIA1 contains 4 Trx domains (named in sequence a-b-b′-a′), in which the redox catalytic domains harboring the CGHC motifs are located in the two a-type domains (a and a′). Catalytic cysteines are shown in black, either reduced (a domain, Cys53 and Cys56) or oxidized (a′ domain, Cys397 and Cys400). Folded PDIA1 assumes a U-shape with both a-type catalytic domains facing each other and the b-type substrate-binding domains (b and b′) forming the bottom of the U. The X-linker, a flexible 19-amino-acid peptide, connects the b′ and a′ domains, conferring high flexibility to PDIA1. The b′ domain contains a hydrophobic binding pocket, which is mainly involved in substrate recognition and binding. The C-terminal portion contains an acidic extension (c domain, not shown), which is involved in calcium binding and exhibits the C-terminal classical ER-retrieval motif KDEL (shown in yellow). (b) Disulfide formation starts with oxidized PDIA1 as a disulfide donor for reduced unfolded client proteins emerging at the ER lumen. A nucleophilic thiolate attack of a substrate Cys on the N-terminal Cys from PDIA1 CGHC motif yields a PDIA1-substrate mixed disulfide. This transient bond is cleaved via the nucleophilic attack of the bound substrate Cys by the resolving substrate Cys, yielding reduced PDIA1 and oxidized substrates. The kinetics of disulfide bond formation are governed by several rate-limiting factors: (1) the deprotonation of Cys thiols to the reactive thiolate species, (2) the steric accessibility of these thiolates to electron acceptors, and (3) the spatial proximity of the participating Cys residues, as dictated by the conformation of the folding intermediate. While the p*K*_a_ of free Cys thiols is approximately 8.7, rendering them largely protonated and unreactive at physiological pH, the local protein microenvironment within a folding intermediate can strongly modulate thiol reactivity. Adjacent electron-withdrawing groups or the α-helix dipole can drop the cysteine p*K*_a_ to values below 5.0, thereby favoring thiolate formation and enhancing reactivity at neutral pH. Furthermore, the process of protein folding, driven by free-energy minimization, brings Cys residues into proximity, facilitating disulfide bond formation. The efficiency of this oxidation is dependent not only on the substrate thiol p*K*_a_ but also on the stability of the disulfide bond in the thiol-oxidoreductase. The disulfide bonds within the active sites of PDIA1 are relatively unstable, making them highly susceptible to reduction by accessible thiols in nascent client proteins. Furthermore, the high p*K*_a_ of PDIA1 C-terminal active site (>10) might prevent it to resolve the mixed disulfide bond which would result in a futile reaction.^83,114–116^ (c,d) PDIA1-mediated reduction targets clients that fail to attain their native disulfides and a fully folded structure, or else as part of repeated oxidation–reduction cycles that correct non-native disulfides (i.e., a “pseudo-” isomerase activity).^117^ Disulfide reduction is also required for retrotranslocation and further degradation in the cytosol during ER-associated protein degradation (ERAD). “True” isomerase activity corrects non-native disulfides in folding intermediates to avoid misfolding and reach native conformations. Disulfide reduction and isomerization start similarly, with reduced PDIA1 and a nucleophilic attack of the N-terminal Cys of PDIA1 CGHC on the substrate disulfide (generally a non-native one), forming a PDIA1-substrate mixed disulfide. When PDIA1 acts as a disulfide reductase, this transient mixed disulfide is attacked by a second thiolate within the PDIA1 active site (C-terminal resolving Cys), generating a reduced substrate and oxidized PDIA1 (c). When PDIA1 acts as a disulfide isomerase, the nucleophilic substrate thiolate generated from the former non-native disulfide upon PDIA1 binding resolves the PDIA1-substrate mixed disulfide in favor of forming a new more stable (native) substrate disulfide, releasing reduced PDIA1. Isomerization involves only one PDIA1 active site, Cys, with no net change in its redox state (d). Created in BioRender.

PDIA1 displays high flexibility, and its intrinsic dynamics helps to adjust its structure to catalyze thiol-oxidoreductase and chaperone activities. Studies using biochemical assays, molecular dynamics, and crystallography showed that reduced PDIA1 is more compact, while oxidized PDIA1 exhibits a relatively open conformation that exposes the shielded hydrophobic pocket in the b′ domain^107,108^ (Figure 2). High-speed atomic force microscopy studies with soluble PDIA1 indicate a high degree of dynamic mobility on a subsecond scale, with higher probability of open configuration for oxidized vs reduced PDIA1.^107^ Importantly, in the presence of more complex substrates, PDIA1 undergoes oligomerization, creating a larger hydrophobic cavity to accommodate the folding client.^107,108^ The main site responsible for such mobility is the X-linker segment, although other sites may also be involved.^109^ This is consistent with reports that chaperone effects, which are supported by b-type domains, are more pronounced upon PDIA1 oxidation, given enhanced exposure of such domains [that is, PDIA1 is a redox-dependent chaperone, although the chaperone effect is not directly redox-related].^110,111^ In contrast, a study using single-molecule fluorescence resonance energy transfer indicates that both redox states show structural heterogeneity, suggesting that PDIA1 adopts multiple conformations in solution.^112^ This indicates that the PDIA1 mobility should be understood as a more dynamic concept than that suggested by a crystallographic snapshot. Of note, as chaperones, PDIs might exhibit chaotropic behavior as a common general property shared with molecular chaperones.^113^

In the ER, PDIA1 catalyzes repeated cycles of thiol oxidation, disulfide isomerization, and reduction to promote proper protein folding. Such reactions involve the differential reactivities of the two Cys of the CGHC motif, as the N-terminal one, given its lower p*K*_a_ assumes the more reactive thiolate form (i.e., deprotonated Cys, S^–^). Such reactions are detailed in Figure 2.

A central feature of the ER redoxome is oxidant generation by the flavoenzyme Ero1 (also termed ER sulfhydryl oxidase 1). Ero1 is present as one (Ero1p) isoform in yeast and two (Ero1α and β) isoforms in human cells. Ero1α is ubiquitously expressed^118^ while Ero1β is enriched in active secretory cells.^119^ PDIA1 is the preferential substrate of Ero1α, although mixed disulfide complexes involving Ero1α and ERp57 and ERp72 have also been reported.^120^ Such a mechanism involves disulfide transfer from the oxidized Ero1α external loop to reduced PDIA1, yielding oxidized PDIA1 plus reduced Ero1α. Reduced Ero1α, in turn, must be reoxidized. Ero1α reoxidation occurs via a two-electron transfer from Ero1α Cys to O_2_ (a universal electron acceptor) via the Ero1α cofactor flavin adenine nucleotide (FAD), yielding H_2_O_2_ (Figure 3). Electron transfer from reduced Ero1α thiols during its reoxidation requires conformational flexibility of its external loop; this mobility is in turn regulated by additional pairs of regulatory Cys distinct from the catalytic ones.^2,121,122^ Under excessively oxidizing ER conditions, such regulatory Cys forms disulfide bonds that restrict loop mobility, thus attenuating Ero1α oxidase activity to halt further H_2_O_2_ generation.^123–125^ Conversely, Ero1 activity requires the regulatory Cys to be reduced, which depends on reduced PDI(s).^126–128^ Details of this interesting protective mechanism have been reviewed previously.^2,129,130^ In addition, Ero1α can be regulated under reductive stress by phosphorylation of Serine-145 in the Golgi, which enhances its oxidase activity.^131^ Ero1α is well-established as being essential for disulfide bond formation, as shown by the lethality of its loss in yeast and plants. However, *ERO1* double mutant mice remain viable, suggesting the existence of compensatory pathways in mammals.^132^ Potential alternative mechanisms include transmembrane ascorbate/dehydroascorbate, vitamin K oxidoreductase (VKOR),^99,133^ GSSG, and H_2_O_2_ itself.^134^ VKOR may prefer oxidation of a specific subset of PDIs such as TMX proteins and ERp18.^135^ Importantly, PDIA1 reacts quite slowly with H_2_O_2,_ with reported rate constants of *k* = 17.3 ± 1.3 M^–1^ s^–1^.^136^ In parallel, ER-resident peroxiredoxin-4 (Prdx4) and ER-localized glutathione peroxidases 7 and 8 (GPx7/8) also serve as electron acceptors for reduced PDIA1 through their fast reaction with H_2_O_2_ and ensuing disulfide transfer to regenerate oxidized PDIA1 ^137–140^ (Figure 3). The rate constants for reactions with H_2_O_2_ are 2.2 × 10^7^ M^–1^ s^–1^ for Prdx4 ^141^ and ca. 10^3^ M^–1^ s^–1^ or lower for Gpx7/8.^138^ Indeed, Prdx4 knockdown in ERO1-double mutant mice led to reductive redox shift and lower cell viability.^137^ Prdx4 effectively oxidizes multiple PDI family members (ERp46, ERp5, ERp57) beyond PDIA1 and its redox state is an index of oxidative stress.^140,142^ Considering the high physiological concentrations of PDIA1 and Prdx4 vs the relatively lower levels of Ero1α,^143^ Prdx4-mediated PDIA1 oxidation is more efficient than that catalyzed by Ero1α. Similarly, GPx7 and GPx8 (a soluble and a type-I membrane protein, respectively) act as PDIA1 peroxidases, coupling the reduction of H_2_O_2_ to oxidize PDIA1.^139^ Overall, while the Ero1α-PDIA1 system generates H_2_O_2_ during protein folding, H_2_O_2_ is productively consumed via the Prdx4-PDIA1 and GPx7/8-PDIA1 systems. There is some evidence suggesting that GPx8 and Prdx4 may target distinct H_2_O_2_ pools, that is, GPx8 preferentially scavenges Ero1α-derived H_2_O_2_, whereas Prdx4 targets H_2_O_2_ from other sources,^137^ although also Ero1α.^142^ Furthermore, GPx7 exhibits higher reactivity with H_2_O_2_ than GPx8, leading to more efficient PDIA1 oxidation^144^ and suggesting it also functions as an H_2_O_2_-dependent PDIA1 oxidase. Together, these data unveil a sophisticated interplay of proteins governing ER H_2_O_2_ pools and indicate that H_2_O_2_ serves a dual role in oxidative folding, acting as both a byproduct and a substrate. Since every introduced disulfide generates one H_2_O_2_ molecule, oxidant flow can be enormous, highlighting the need for tight control of such counterbalancing mechanisms to sustain adequate ER redox balance.^30^

**Figure 3:**
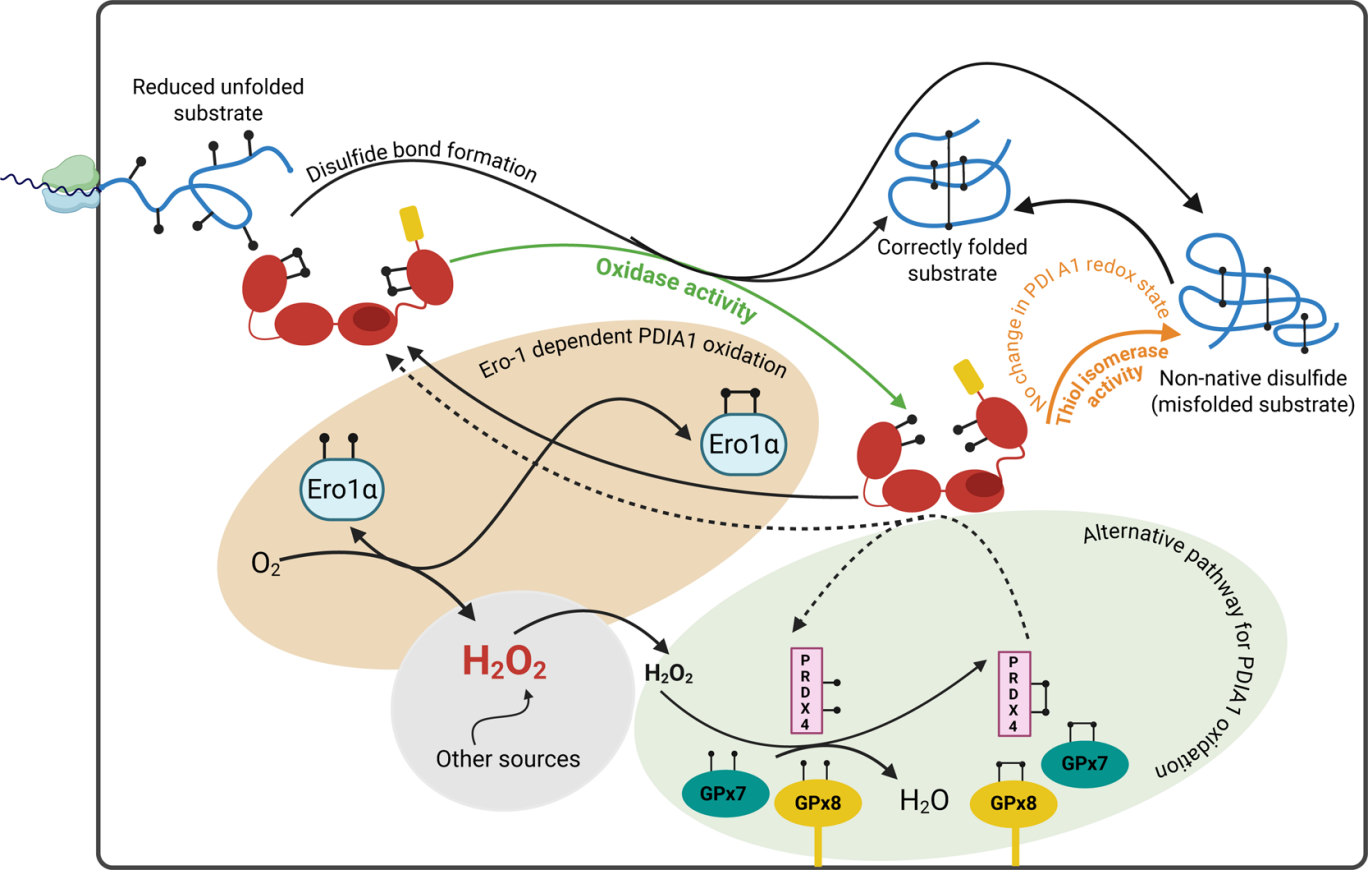
Overview of oxidative folding pathways in the ER. Nascent client proteins enter the ER via cotranslational translocation in an unfolded state, with Cys residues present as free thiols. Within the ER, oxidative protein folding facilitated by PDIA1 then catalyzes the formation and rearrangement of disulfide bonds, which are essential for these proteins to achieve their native conformations. The process is initiated by oxidized PDIA1, which exhibits a higher probability of open conformations and a catalytic disulfide. In this case, PDIA1 acts as an oxidase (green line), donating its disulfide to reduced substrate proteins. The outcome of this transfer is the formation of folding intermediates possessing either native or non-native disulfide bonds. Concomitantly, PDIA1 is left reduced, prompting a conformational shift to a higher probability of a closed state with dithiol motifs. Reduced PDIA1 supports the rearrangement of non-native disulfides into their correct form, i.e., thiol isomerase activity (orange line). Such isomerase activity is essential for generating the native disulfide pairings required for the correct protein folding. PDIA1-induced isomerization starts and ends with reduced PDIA1. Oxidized PDIA1 is regenerated primarily by Ero1α, which consumes molecular oxygen and produces H_2_O_2_ as a byproduct. In parallel, PDIA1 can be reoxidized through a peroxide-dependent pathway mediated by Prdx4 and/or GPx7/8 (light green circle; dashed lines). Thus, Prdx4 and Gpx7–8 functions are efficiently funneled toward PDIA1 reoxidation, optimizing the rates of disulfide bond generation and productively using H_2_O_2_ generated by Ero1α and potentially other sources. Thus, H_2_O_2_ is both generated and consumed during disulfide formation. Created in BioRender.

In contrast to the PDI-dependent mechanisms described so far, quiescin sulfhydryl oxidase (QSOX) can directly oxidize clients without relying on PDIs.^7,30,99,130^ However, QSOX does not isomerase non-native disulfides; thus, native disulfide formation is enhanced in the presence of PDIA1.^145^ QSOX effects rely on its dual-domain architecture, combining a flavoenzyme domain homologous to Ero1 with a Trx domain similar to that of PDIs. Electrons derived from the polypeptide substrate are accepted by the Trx domain and subsequently transferred to FAD via two internal disulfide exchange reactions.^99,130^ Indeed, QSOX might fulfill some Ero-1 functions in its absence.^146^ Interestingly, in vitro QSOX does not significantly oxidize reduced PDIA1, consistent with QSOX performing a targeted oxidation of reduced substrates without affecting PDIA1 pools.^145^ However, the contribution of QSOX to the global disulfide output of the mammalian ER remains unclear. Importantly, QSOX is primarily located in the Golgi apparatus^146,147^ being also secreted by cells.^147^ This suggests that disulfides can be formed in extra-ER sites along the secretory pathways or extracellularly.

Hydrogen peroxide is a physiological oxidant with central roles in biological redox signaling.^148,149^ Local H_2_O_2_ concentrations in the ER lumen and other cellular compartments are both actors and reports of their respective redox signaling networks. While H_2_O_2_ can diffuse rapidly across membranes, including the ER membrane,^150^ the maintenance of sharp H_2_O_2_ gradients among distinct organelles (Figure 1) strongly argues for its regulated transmembrane movement, known to be provided by specialized aquaporin (AQP) channels—termed peroxiporins.^151^ Notably, the ER-localized mammalian peroxiporin AQP11 facilitates efficient H_2_O_2_ transport across the ER membrane^152^ and regulates the transfer of mitochondrial-derived H_2_O_2_ to the ER.^153^ Plasma membrane peroxiporins (AQP3, 8 and 9) also interact with the ER at ER contact sites,^154^ as discussed below. ER lumen H_2_O_2_ levels were reported as ca.700 nM, potentially increasing to ∼1.26 μM upon stimulation (ratiometric probe α-Naph).^155,156^ Indeed, oxidative protein folding renders the ER lumen a more potent raw source of oxidants, for instance, than mitochondria, as reported in liver homogenates (42 vs 12 nmol/min/g liver tissue in the ER microsomes vs mitochondria, respectively). Such studies suggest that the ER contributes to 45% of total cellular H_2_O_2_ production vs only 15% for mitochondria.^157–159^ Mitochondria levels are estimated to be < 4 nM (kinetic model).^156,160^ Furthermore, a peroxide-responsive biosensor targeted to the ER (HyPer-ER) becomes mainly oxidized, further stressing a highly oxidizing ER environment.^161^ However, HyPer-ER oxidation is debatable as a proxy of H_2_O_2_ production as it is a disulfide-based sensor targeted to a site in which thiol oxidoreductases are highly enriched and may directly oxidize the sensor disulfide, confounding interpretation.^162,163^ Beyond oxidative folding, there are other sources of H_2_O_2_ in the ER such as Nox4 and microsomal cytochrome P450.^164^ Nox4 is predominantly localized to the ER but is also detectable in other subcellular compartments.^165^ Unlike other Nox isoforms, Nox4 is constitutively active, generating mainly H_2_O_2_ rather than superoxide (such as Nox1, e.g.), suggesting its potential physiological roles in ER redox homeostasis under specific conditions.^165^

In contrast to ROS sources, current knowledge about ER-specific antioxidant systems is limited. Notably, the ER exhibits a markedly low abundance of “classical” antioxidant enzymes, with undetectable or minimal levels of SOD1, catalase, and glutathione reductase. Furthermore, TrxR and Trx have not been identified in the ER (see below). Several PDIs with reductase activity are located at the ER; however, their potential antioxidant defense roles are unclear.^166^ In this regard, the peroxidases GPx7/8 and Prdx4 stand out as mechanisms to control ER H_2_O_2_ levels.^167,168^

#### Reductive Pathways

ER reductive pathways are as critical as oxidation during protein folding, as disulfide reduction is essential to correct non-native disulfides, to allow ER-associated protein degradation (ERAD), to govern the UPR, and to regulate protein functions^100,168–170^ (Figure 4). As discussed above, PDI family members keep their active sites reduced in order to catalyze disulfide reduction and isomerization. Yet, how PDI active sites are reduced within the ER is not clear-cut. In the cytosol, the main disulfide reductase, Trx, is maintained in a reduced state by TrxR, which uses NADPH as electron donor (Figure 4). Similarly, glutathione reductase uses NADPH to convert GSSG into GSH, sustaining the cytosolic glutathione redox buffer.^171^ In contrast, the ER lacks a dedicated reductase for PDIs analogous to TrxR, despite the presence of NADPH (generated by the enzyme hexose 6-phosphate dehydrogenase) and an ER-localized glutathione reductase homologue—ER flavoprotein associated with degradation, ERFAD—which lacks the catalytic cysteines present in the cytosolic enzyme.^172,173^ Instead, the GSH/GSSG redox buffer plays a pivotal role in keeping PDIs reduced,^130,174^ allowing their reductase and isomerase activities. Indeed, most PDIs stay predominantly reduced in the ER,^30,175^ suggesting that disulfides formed within these proteins are rapidly exchanged with either client substrates or GSH to form GSSG.^140^ In fact, GSH efficiently reduces PDIs such as PDIA1 and ERp57, along with evidence supporting that cytosolic NADPH regeneration is essential for proper disulfide formation in the ER lumen.^176^ This implies that cytosolic reductive pathways may regulate the ER luminal disulfide chemistry. Analogous pathways exist in prokaryotes: in the bacterial periplasm, disulfides are introduced into client proteins by DsbB/DsbA (oxidative pathway) and corrected by DsbC/DsbG (isomerases and reductases). These are in turn reduced by the membrane-embedded reductase DsbD, which is kept reduced by cytosolic Trx/TrxR, with NADPH as an electron donor.^100,177^ Recent observations^178^ employing ER-derived microsomes and in vitro reconstituted Trx/TrxR suggested that a membrane-embedded protein is required to mediate reductive transfer across the ER membrane, likely through a mechanism analogous to the bacterial DsbD system (Figure 4). The identity of the membrane protein is unknown, but the lipase maturation factor 1 (LMF1) is a potential candidate.^178^ These data further stress that the Trx/TrxR system would be sufficient to supply reducing equivalents for reducing both regulatory and non-native disulfides in ER-resident proteins, including Ero1α and VKORC1L1.^178^ In some cases, however, GSH-independent pathway(s) may also support ER protein reduction,^100,178^ e.g., the ER reductase ERdj5 and other oxidoreductases.

**Figure 4:**
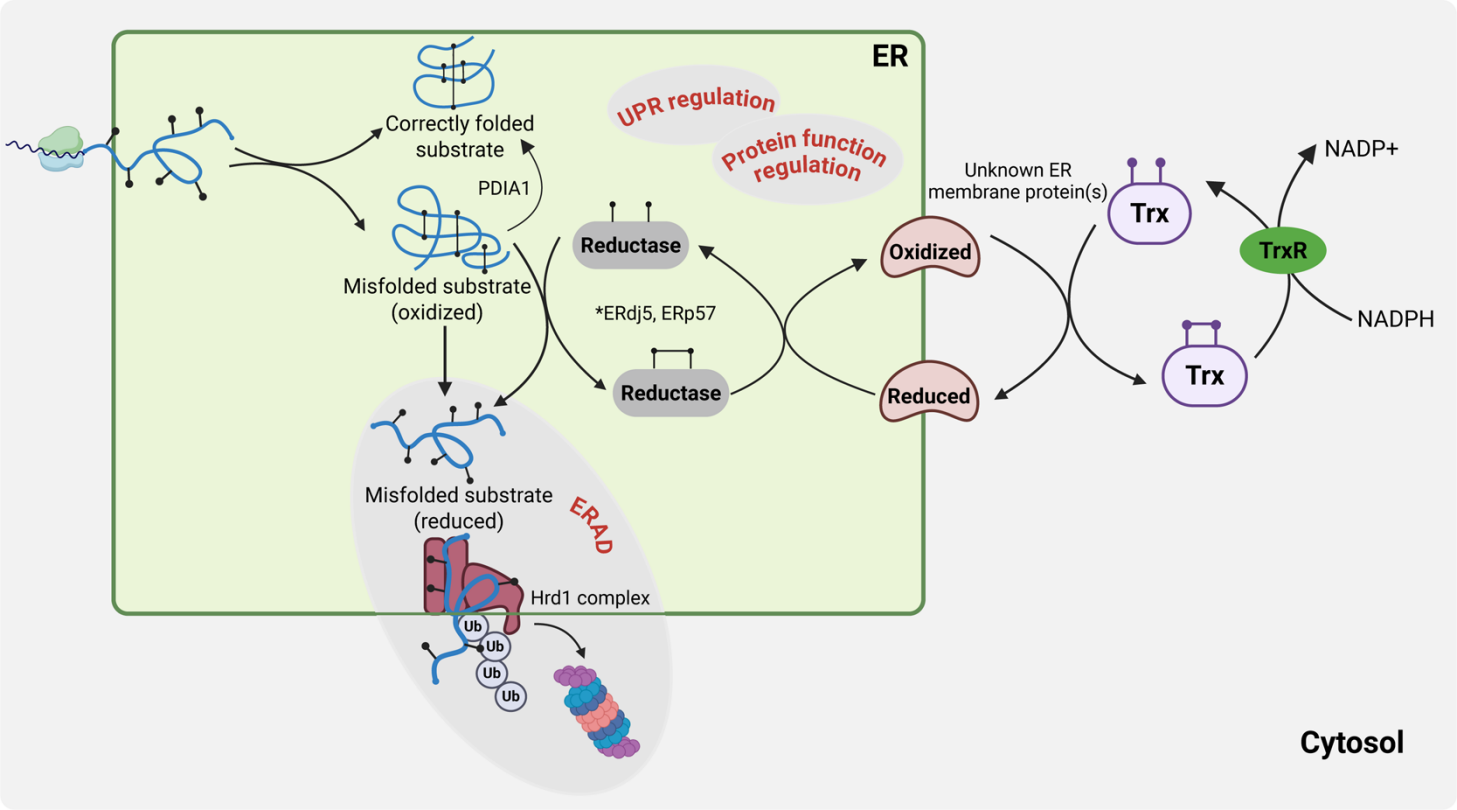
Schematic overview of ER reductive pathways. The ability of PDI family members to catalyze both disulfide formation and reduction requires their active sites to cycle between oxidized and reduced states. Therefore, the ER must maintain pathways for not only oxidizing PDIs but also reducing them. Reduced PDIs are able to cleave non-native disulfide bonds within un/misfolded substrates via a reductive pathway ultimately fueled by cytoplasmic thioredoxin (Trx)/thioredoxin reductase (TrxR) systems. This pathway uses NADPH as its primary electron donor, generated, for instance, by glucose 6-phosphate dehydrogenase. TrxR1 then employs NADPH to reduce Trx. The mechanism by which reduced cytosolic Trx couples to the reduction of ER-localized oxidoreductases, such as ERdj5 and PDIA3, is not fully understood. However, it is likely mediated by currently unknown ER membrane protein(s) able to transfer reducing equivalents across the ER membrane, similar to mechanisms found in prokaryotes. A reductive pathway in the ER is crucial for correct folding and for the degradation of misfolded proteins via ERAD (cytosolic proteasome degradation) as well as UPR and protein function regulation (details discussed in the text). Created in BioRender.

A paradigm-changing recent development is the finding that enhanced protein folding occurs via Ca^2+^-dependent liquid phase separation of ERp5 condensates in the ER lumen, while other PDIs (PDIA1 and Erp57) appear less efficient for this mechanism.^179^ In parallel, another report also showed that Ca^2+^-dependent protein condensates centered around ERp5 and containing PDIA1, Grp78, Grp94, and ERdj3 may accelerate protein folding in the ER lumen.^180^ These observations bring a novel level of understanding to how proteins achieve their native configuration in the ER lumen.

## ER Stress, UPR, and ERAD: Redox Connections

The ER protein-folding capacity varies substantially across cell types; e.g., the ER is expanded in secretory cells to accommodate their high demands for protein secretion. Despite an overall robust machinery for ER protein folding/processing, many client proteins still do not achieve their native states and are degraded, indicating a somewhat low folding efficiency within the secretory pathway.^6,181^ Given the significant cellular toxicity of un/misfolded proteins, cells have developed elaborate quality control mechanisms to prevent and/or dispatch them, including the *ERAD* pathway and the UPR. The former allows incompletely folded proteins to retrotranslocate to the cytosol followed by ubiquitination and proteasomal degradation,^182^ while the latter is a restorative/adaptive stress response.^83,183,184^ Several physio­(patho)­logical conditions overcharge the ER, such as nutrient deprivation, hypoxia, impaired Ca^2+^ homeostasis, glycosylation dysfunction, or expression of mutated secreted proteins, which generated folding-defective species and disrupted ER redox homeostasis associated with ROS overproduction.^168^ These conditions are characterized by imbalances between protein-folding demand and protein-folding capacity, causing the accumulation of unfolded and misfolded proteins in the ER, disrupting its normal physiology. This state is defined as ER stress, which leads to activation of the UPR signaling pathway to restore ER homeostasis. UPR signaling has been addressed in excellent reviews and will not be focused in depth here.^83,181,184–186^ Briefly, the UPR comprises signaling from three classes of ER stress transducers, each defining a distinct arm. Such sensors/transducers consist of two ER transmembrane protein kinases, the inositol-requiring protein-1α (IRE1α) and the kinase RNA (PKR)-like ER kinase (PERK), as well as activating transcription factor-6 (ATF6). They function independently but communicate extensively.^183,187^ In each case, they sense the protein folding status in the ER lumen and transmit this information across the ER membrane to the cytosol. Comprehensive reviews outline key mechanisms of UPR activation.^184,186,188^ Under physiological conditions, IRE1α, PERK, and ATF6 remain inactive due to their luminal domain association with Grp78. Once unfolded or misfolded proteins accumulate in the ER, Grp78 preferentially binds to them and dissociates from UPR transducers, leading to their activation and downstream signaling. Activation of the three UPR arms triggers cellular events to mitigate protein un/misfolding, which include attenuation of protein synthesis, degradation of un/misfolded proteins, and a massive upregulation in the proteostasis transcriptome (protein folding, translocation, and degradation) to promote cell survival. UPR activation also triggers pro-apoptotic signal cascades especially under prolonged ER stress, when homeostatic UPR signaling is exhausted (maladaptative UPR), resulting in ER stress-induced cell death.^181,189^

ER stress and UPR activation closely associate with ER redox homeostasis and ROS may function as UPR signaling molecules, though which ROS is produced in this case is not clear-cut.^166,190^ Although ROS increases per se can cause ER stress and UPR signaling, this has not been systematically addressed and appears to depend on context and cell type.^168,191^ The age-dependent shift toward a less oxidizing ER, coupled to less reducing cytosol,^192^ associates with impaired protein folding, ER stress susceptibility, and maladaptive UPR.^193^ As discussed above, the Ero-1-PDIA1 axis, crucial for protein folding, needs a tight balance to maintain optimal proteostasis. Indeed, maladaptative ER stress associates with abnormal ER H_2_O_2_ generation, which can further disrupt oxidative folding. CHOP (C/EBP homologous protein), an ER stress-induced transcription factor (PERK arm), can promote a hyperoxidizing ER through Ero1α upregulation, aggravating protein misfolding.^194,195^ In contrast, GPx8, which is induced upon ER stress, can control Ero1α-derived ROS and cell survival.^167^ Also, PERK activation stimulates several antioxidant defenses via the activating transcription factor (ATF4) and/or via NRF2/Keap.^166^ Another important ER source of ROS during ER stress is Nox4, which exhibits increased expression and activity upon tunicamycin-induced ER stress in vascular smooth muscle cells.^166^

Although Grp78 is the primary allosteric regulator of the three UPR transducers, changes in the ER oxidative state can also directly activate IRE1α, PERK, and ATF6 signaling in a PDI-related manner. PDIA5 (PDIr) can promote thiol–disulfide reshuffling in ATF6 under ER stress, leading to its ER-Golgi export and consequent activation.^196^ Also, ERp5 binds to an oxidized Cys residue from luminal IRE1α domain, both suppressing its activation and facilitating its return to inactive conformation.^197^ Moreover, ERp5-IRE1α axis is controlled by micro-RNA-322 after ER Ca^2+^ depletion-induced ER stress.^198^ ERp5 also functionally associates with PERK via thiol–disulfide exchange.^185,197^ In addition, PDIA1 is phosphorylated by FAM20-C under ER stress, switching function from a foldase to a holdase, which prevents protein misfolding and directly binds IRE1α in a Cys-independent fashion, attenuating its activity.^199^ IRE1α redox regulation also plays roles in UPR antioxidant responses. Sulfenylation of a conserved Cys in IRE1α inhibits its kinase activity and attenuates NRF2/SKN-1-dependent antioxidant response.^200^

ER luminal redox changes also affect Ca^2+^ homeostasis, which connects to ER stress responses, while ER Ca^2+^ efflux is a hallmark of ER stress.^168^ Decreases in ER Ca^2+^ levels upon ER stress associate with SERCA inhibition and passive release by IP3R. Additionally, oxidation of specific Cys residues in Ca^2+^ channels can induce Ca^2+^ release from the ER^189^ (see below). Conversely, the PDI family member ERp44 interacts with IP3R1 luminal domain Cys residues when these are reduced, inhibiting IP3R1-dependent Ca^2+^ release.^201^ Importantly, loss of ER Ca^2+^ homeostasis impairs the activity of Ca^2+^-dependent ER chaperones and potentiates ER stress.^85^ In addition, short-term ER Ca^2+^ depletion leads to decreased ER redox potential (i.e., ER lumen reductive shift of 24 mV).^202^ However, other strong and sustained ER stressor stimuli do not elicit similar reducing shifts and may not affect steady-state ER thiol redox poise,^202^ contrary to ER-stressed yeast, which exhibit an underoxidized ER.^203^ These observations indicate that mammalian cells defend their ER redox poise quite efficiently during severe ER stress.^202^ Potential mechanisms for ER hypo-oxidation upon Ca^2+^ depletion may involve PDIA1 binding to CRT, resulting in PDIA1 sequestration and attenuation of its oxidative ER effects^204^ and the ER influx of cytosolic GSH.^205^ In summary, interactions between the ER redox and Ca^2+^ signaling are critical determinants of ER stress responses.

Although the ERAD pathway is constitutively active, the UPR transcriptionally induces several ERAD components. Constitutive ERAD degrades unfolded, misfolded or even natively folded proteins. This process not only ensures proteostatic fidelity but also regulates the abundance of specific protein substrates.^206,207^ IRE1α itself is an endogenous substrate of Sel1L-Hrd1-mediated degradation, which supports constitutive ERAD. Its constitutive degradation serves as a critical mechanism to restrain IRE1α signaling activity under basal physiological conditions in diverse cell types.^206,208^ Additional mammalian proteins regulated in this way include IP3R, β-catenin, and apolipoprotein B (ApoB). ERAD induction under ER stress occurs via both ATF6 and IRE1α via spliced XBP1-mediated increase in the expression of genes encoding the Hrd1 complex, which supports retrotranslocation of luminal substrates from ER to the cytosol for proteasomal degradation.^209–212^ Several studies established a direct connection between redox processes and ERAD, highlighting the importance of redox pathways in the recognition and disposal of misfolded proteins. The primary and most well-established link involves PDIs, which mediate the reduction of misfolded proteins (via the reductive pathway described earlier) as a critical step enabling their recognition and subsequent degradation by ERAD. In parallel, PDIs such as PDIA1, TXNDC11, ERp46 and ERdj5 may form complexes with lectins from ERAD machinery through intermolecular disulfide bonds, regulating their activity.^212^ Recently, yeast Pdi1 was shown to act as a reductase in the Mnl1–Pdi1 ERAD complex. This complex initiates ERAD in yeast, mediating glycan trimming of misfolded globular proteins and subsequent disulfide bond reduction. While Pdi1 within the complex loses its canonical oxidative activity, it retains its disulfide reductase functionality. Notably, the association between Mnl1 and Pdi1 does not impair its isomerase activity. The resulting unfolded polypeptides are then retrotranslocated from the ER to the cytosol for degradation, clearly reinforcing that Pdi1 is the long-sought ER reductase for ERAD.^213^

## ER and Its Membrane Contact Sites: Hubs for Redox Communication

The ER establishes membrane contact sites with essentially all other cellular organelles, such as plasma membrane (ER-PMcs), mitochondria (mitochondrial-associated membrane termed MAMs), peroxisomes (ER-POcs), Golgi, endosomes, and lipid droplets, among others. These ER contact sites consist of synapse-like regions in which membranes of the ER and another organelle come into close proximity, usually ca. 10 to 30 nm, without fusing^96^ and may involve in some cases liquid phase separation.^214^ ER membrane contact sites are essential for maintaining ion and lipid homeostasis,^96^ regulating signaling pathways, and managing organelle dynamics, such as mitochondrial fusion/fission through Mitofusin2.^96^ Such abundance of contact sites aligned to the roles of ER in endomembrane traffic, with the ER contributing to ca.50% of the total membrane pool of mammalian cells.^215^ ER contact sites are stabilized by specific tethering proteins and ion channels, mainly for Ca^2+^, enabling direct communication and interorganelle transfer of ions, lipids, and small molecules such as H_2_O_2_. In addition, increasing evidence implicates these contacts as hubs for redox communication and signaling. A “redox quadrangle” comprising ER-plasma membrane-mitochondria and peroxisome membrane contacts has been recently conceptualized to modulate cellular redox regulation.^149,216,217^ Here, we highlight some aspects of such redox regulation (Figure 5).

**Figure 5:**
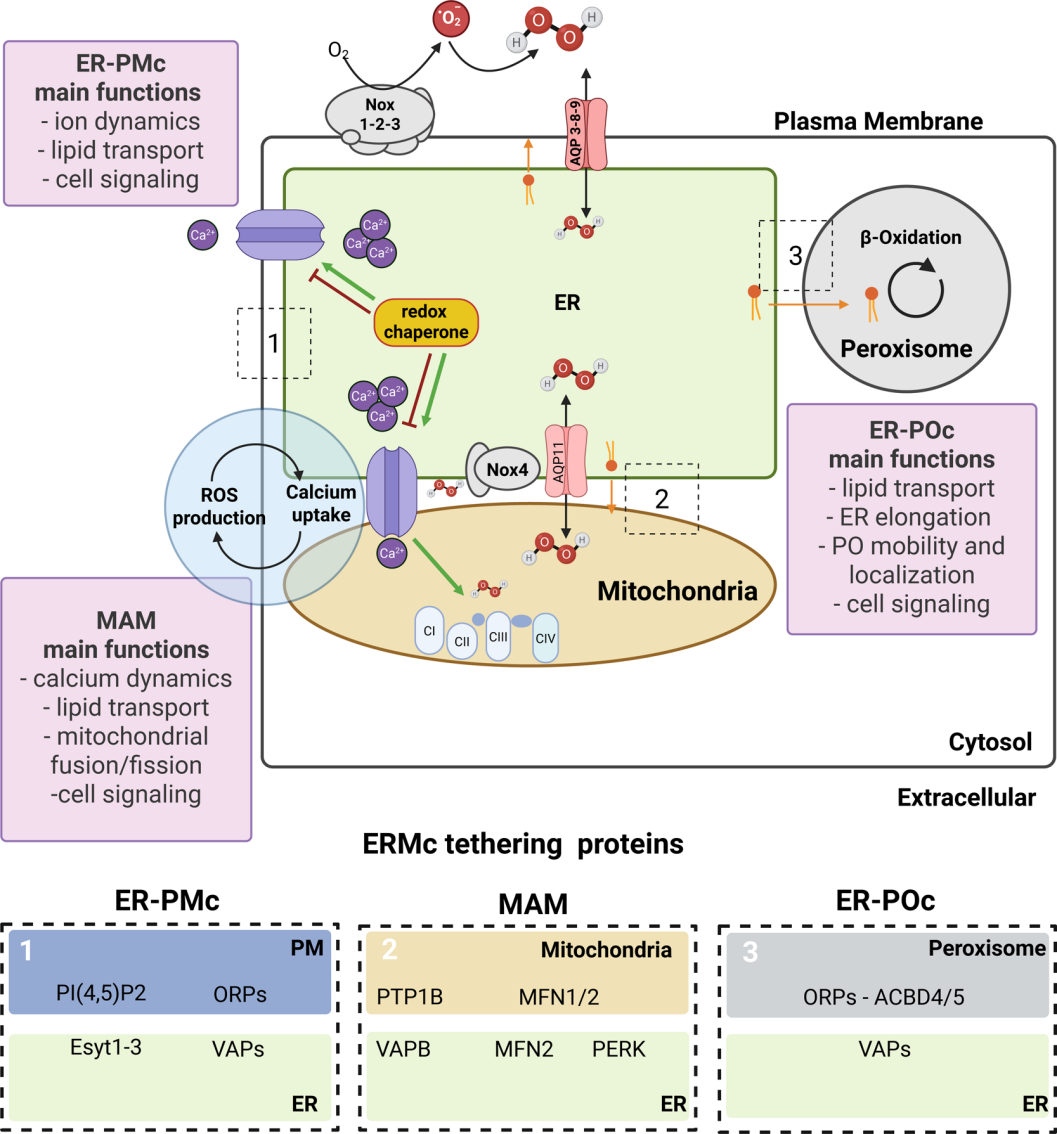
Schematic overview of redox-related ER membrane contact sites (ERMCs). ER membrane contact sites play a central role in maintaining ion and lipid homeostasis, as well as regulating organelle localization, translocation, and dynamics, including peroxisome and mitochondrial fusion/fission events. These contact sites, as key modulators of cell signaling, are also involved in redox signaling. ER Ca^2+^ signaling and redox homeostasis are tightly interconnected: ER-mediated Ca^2+^ fluxes promote ROS production, while ROS can modulate the Ca^2+^ channel activity. In addition, ER-resident redox chaperones also regulate Ca^2+^ channels in a redox-dependent manner. ER membrane contact sites are also involved in direct interorganelle transport of H_2_O_2_ via aquaporins. Besides Ca^2+^ channels, several other tethering proteins at the ER contacts are subject to redox regulation (see insets 1–3). Created in BioRender.

### Hydrogen Peroxide

ER membrane contacts can be a preferential site for production and transport of H_2_O_2_, generated by sources such as Nox4, Ero1α, and mitochondria. Indeed, MAMs has nanodomains of H_2_O_2_,^218^ which is transported across membranes via local AQP11.^152^ Forced decrease of ER H_2_O_2_ levels promoted by Ero1α downregulation is compensated by the transfer of mitochondrial complex III-derived H_2_O_2_ to the ER via AQP11. Similar transport through AQPs was also described in plant ER-PMcs.^154^ Data from our group (DeBessa et al., unpublished observations) indicate that ER-PMcs are hubs for Nox1 NADPH oxidase and PDIA1 can reshape ER-PMcs organization.

### Tethering Proteins

The ER redox state directly influences ER membrane contacts via conformational changes in tethering proteins, potentially affecting their interactions or lipid transfer between membranes.^216^ An extensive proteomic analysis of potentially redox-regulated proteins based upon Cys reactivity^219^ unveiled several hits related to ER tethering proteins of membrane contact sites. These include vesicle-associated membrane protein (VAMP)-associated proteins (VAPs), which are ER-tethering proteins present in ER-PMcs, MAMs, and ER-POcs and bind to partners such as protein-tyrosine phosphatase 1B, oxysterol-binding protein-like 10, reticulons, and kinesin. VAP A and B tether ACBD4/5 peroxisomal proteins to compose ER-POcs in the course of β-oxidation and ER-peroxisome lipid transport.^220,221^ Disruption of ER-POcs (ACBD5/VAP) enhances peroxisome mobility.^222^ Interestingly, Ceapin ATF6 inhibitors induce ER-POcs by tethering ATF6 cytosolic portion to ACBD3 transmembrane regions.^223^ Beyond its role as an UPR sensor, PERK was also identified as a MAM-tethering protein as the silencing of PERK or Mitofusin2 (an upstream PERK regulator)^224^ leads to decrease in MAMs, ROS deregulation, and apoptosis.^225,226^ PERK can also coordinate ER-PMc formation through filamin-A and F-actin remodeling.^227^

### Calcium Channels

Ca^2+^ channels play important roles as anchoring proteins in the ER membrane contact dynamics and stabilization. Redox signaling at ER membrane contacts is tightly integrated with Ca^2+^ dynamics, especially at ER-PMcs and MAMs, in line with the already discussed convergence between ER redox potential and ER Ca^2+^ levels.^228^ At ER-PMc, STIM and ORAI (calcium-release-activated calcium modulators), key components building the SOCE channel from the ER and the PM side, respectively, are redox-modulated. The luminal Cys residues (Cys49 and Cys56) of STIM1 were implicated in redox sensing within the ER lumen^229^ and interact with PDIA1 and ERp57. PDIA1 or ERp57 loss of function promotes enhanced SOCE amplitude under partial store depletion, by fine-tuning STIM1 Ca^2+^ binding affinity.^230^ In platelets, Ero1α loss of function impairs its redox-dependent interaction with STIM1 Cys49 and 56 and decreases platelet activation/aggregation.^231^ Oxidative stress can promote STIM1 oligomerization and SOCE activation as follows. Cys56 can be *S*-glutathionylated in response to oxidant stress, leading to constitutive Ca^2+^ entry.^232,233^ STIM1 redox-dependent conformational change promotes its translocation to ER-PMc to activate ORAI channels. In turn, cytosolic ORAI1 Cys195 oxidation increases its activity in response to oxidative stress,^229^ while ORAI3, which is devoid of this Cys, is not responsive to oxidative stress. Another redox-sensitive target is the ER-localized IP3R, which is significantly enriched at MAMs and ER-PMcs.^229^ H_2_O_2_ nanodomains at MAMs promote IP3R-mediated Ca^2+^ release.^234^ Also, ROS enrichment in MAMs increases Grp78-mediated IP3R binding to voltage-dependent anion channel and the ensuing Ca^2+^ entry at MAMs further upregulates ROS generation.^235^ ER redox regulates IP3Rs by targeting mainly two Cys atoms critical for the assembly of functional tetramers. Oxidation of another Cys pair by ERp46 or Ero1α further supports IP3R activation, while their reduction by ERdj5 promotes IP3R inactivation.^236^ PDIA1 can also indirectly play a role in IP3R activation.^236^ In stressed cardiomyocytes and fibroblasts, Nox4 redox-regulates IP3R at MAMs, inhibiting Ca^2+^ release into mitochondria and preventing mitochondrial permeability transition and necrosis.^237^ Overall, these examples highlight patterns of how ER redox regulates membrane contact dynamics and, conversely, how ER membrane contacts regulate redox state of the ER and other organelles via Ca^2+^ signaling, ROS production, and transport (Figure 5).

## Signaling Targets and Biological Effects of ER-Dependent Outreach Redoxome

In parallel with aspects of the ER redoxome discussed so far, there is increasing evidence for regulation of extra-ER signaling targets mediated by ER-based oxidoreductases and possibly by ER-derived oxidants, with specific signaling targets.^4^ Here, we collectively refer to such pathways as the ER-dependent outreach redoxome (ERDOR), although some of these effects may not be directly redox-mediated. A key component of ERDOR are the ER thiol oxidoreductases, mainly from the PDI family, as well as Ero1α. Here, we focus our discussion on less appreciated general mechanisms of ERDOR, while general organization and physiological effects were previously addressed.^2,3,7,30,238^ ERDOR effects can be mediated under 3 general modes: (1) sources of oxidants leaking from the ER; (2) thiol redox actors belonging to extended or translocated ER-derived structures; or (3) thiol redox effects of their direct translocation, particularly extracellularly. Before we advance into this discussion, it is important to review some basic aspects of the PDI redox code as follows.

## The PDI Redox Code

The evident roles of PDI family members in the outreach ER redoxome indicate that PDIs and related oxidoreductases should be included in the category of professional thiol redox proteins composing overall cellular, not only ER-specific, thiol redox code.^238^ It is important, however, that the “PDI redox code” substantially differs from that of other professional thiol redox proteins, such as, e.g., the peroxiredoxins.^239^ A major difference lies in the slow rate of reaction with small-molecule oxidants, low for PDI and high for Prdx(s).^12,136,238^ Moreover, the concentrations of PDIs outside the ER are in general much lower vs those of Prdxs, possibly even in the EC milieu.^4^ Thus, any roles of PDIs in physiological redox-dependent signaling should involve preferential acceleration of thiol–disulfide exchange reactions in focused targets. In fact, thiol oxidoreductases in general may accelerate thiol–disulfide exchange in client proteins up to ca. 10^4^-fold.^156,240^ In contrast, their thiol oxidation by small molecule oxidants is kinetically disfavored, unless insulated within specific compartments. In this regard, enrichment of PDIs in small subcellular domains (e.g., vesicles or endosomes)^241^ may partially offset their low levels and enhance the chance of thiol oxidation reactions. This aligns with common strategies used by cells to focus kinetically unfavorable reactions to specific protein targets, as for ex. the usual slow reaction of thiol-proteins with H_2_O_2_.^239,242^ These considerations suggest that the PDI-related outreach redoxome behaves neither as a mass peroxide sensor system nor as a direct effector of global cellular redox state regulation such as the Prdxs. Rather, their effects associate with specific focused targets and are likely influenced by a local rather than global thiol redox state. From a physiological standpoint, PDIs are unlikely to mediate housekeeping redox signaling as Prdxs do. Rather, they tend to support fine-tuning of cellular and tissue remodeling, while signaling specificity can be achieved by focused target localization plus kinetic acceleration of thiol–disulfide exchange. As a consequence, the PDI redox code is likely to be governed by two important factors: the subcellular location and molecular interactors.

Although in most cells and tissues PDIs seem to be kept as a mixture of oxidized and reduced forms,^2,110,116,243^ a particular feature of the PDI code within ERDOR, versus their roles within the ER, may be thought of as an enhanced regulation by substrates. This is due to widely different PDI/substrate ratios in the ER (high) vs other locations (low). In the ER lumen, the upper micro to millimolar PDI levels^244^ highlight the redox state of PDIs as a reflection of their dominant regulation of substrate redox, while at other locations, e.g., EC, the substrates may to some extent dictate PDI redox state. For this reason, the redox state of PDIs closely depends on the physiological context, subcellular location, and cell type.

The PDI redox code also comprises a number of redox post-translational modifications beyond the classical thiol–disulfide formation discussed so far.^2,3,102^ These can help modulate the conformational structure, type of activity, subcellular location, and functional implications of PDIs and were mostly described for PDIA1. PDIA1 sulfenylation^245,246^ has been increasingly recognized and is able to enhance platelet activity and thrombosis caused by oxidized LDL. ERp57, but not ERp5, is also sulfenylated under these conditions. In endothelial cells, PDIA1 sulfenylation is enhanced by VEGF during angiogenesis.^241^ In contrast, PDIA1 *S*-nitrosation inhibits platelet aggregation.^247^ PDIA1 *S*-nitrosation has been proposed to abrogate its protective effects against neurodegeneration.^248^ PDIA1 *S*-glutathyonylation, occurring under oxidative conditions via glutathione transferase-π1, impairs its isomerase and chaperone activities and drives PDIA1 to the ER-mitochondria interface, contributing to apoptosis.^249^ *S*-nitrosation and glutathyonylation appear inter-related since exposure to NO donors can promote PDIA1 *S*-glutathyonylation together with ER stress^250^ and, conversely, glutathione depletion promotes PDIA1 *S*-nitrosation in neuroblastoma cells.^251^ PDIA1, as well as ERp57 and ERp72, are potential targets of persulfidation,^252^ while the physiological implications of this PTM require further investigation. Importantly, some of these redox PTMs target catalytic Cys (e.g., sulfenylation of a-domain Cys in platelets),^245^ while in other cases PTMs (e.g., sulfenylation, persulfidation) are reported in noncatalytic Cys312 or Cys343.^252,253^ Other PDIA1 PTMs are not directly redox-related but may affect redox modifications. These include PDIA1 phosphorylation, discussed above, which occurs during ER stress and induces a more open protein conformation,^199^ as well as succinylation, O-Glcnacylation,^3^ and ubiquitination. N-terminal arginylation of Grp78 and PDIA1 is coinduced and coordinates autophagy via associated p62.^254^ Moreover, forced deficiency in PDIA1 UFMylation promotes its degradation and mitochondrial dysfunction.^255^ Although only a few PDIs display glycosylation motifs, ERp57 can bind to glycosylated CNX and migrate to surface of tumor cells, where this complex promotes reduction of several extracellular matrix (ECM) protein disulfides.^256^

Another emerging PDI code component is the increasingly evident asymmetry of the protein molecule, typified by the distinct features of the a and a′ domains of PDIA1, as well as potentially other PDIs having 2 or more a-type domains. Such asymmetry fits with the known evolutionarily acquired interdomain cooperation within PDIs, opposed to the individuality of Dsb functions in prokaryotes.^56^ While the two PDIA1 a-type domains depict similar redox potentials and active site sequences,^257^ the a domain preferentially undergoes sulfenylation and exerts regulation of thrombosis associated with oxidative events.^245^ The a, but not a′, domain is essentially required for survival, while agonist-induced thrombosis reportedly associates mainly with the a′ domain.^258^ The a′ domain uniquely binds Ero1α and Prdx4,^259^ while the a domain may also have disaggregase functions.^106^ Importantly, in basal conditions, the a domain is detected as partially reduced, while a′ domain is 80% oxidized.^245^ The asymmetry of a′ vs a domains may cause secondary rearrangements of b-type domains, affecting other PDI properties. This asymmetry can also explain experimental findings of partial oxidized states and simultaneous oxidase and isomerase activities for PDIA1.^245,246^ Overall, the asymmetric PDI configuration likely contributes to widening the spectrum of their distinct functions.

### ERDOR via ER-Derived Products

ERDOR may potentially involve the passive or active overflow of ER-generated low-molecular weight oxidants (mainly H_2_O_2_), via ER membrane-specific AQP11 channels.^152^ In fact, AQP11 silencing perturbs the biogenesis of polycystin-1 in a way reversible by ER-targeted catalase,^260^ suggesting a normal outward flow of ER-derived H_2_O_2_. This H_2_O_2_ may particularly affect ER-adjacent targets such as the cytoskeleton,^261^ however this remains speculative. Outward Ca^2+^ flux to the cytosol is another potential ERDOR regulator, with substantial information implicating the translocon pore Sec61 as an emerging relevant pathway for Ca^2+^ leakage into the cytosol during ER stress and other conditions.^262^ Sec61B was shown recently to mediate platelet activation in diabetes via enhanced Ca^2+^ flux into the cytosol.^263^

### ERDOR via Extended/Translocated ER-Derived Domains

Enrichment of ER oxidoreductases within specific ER-derived microdomains may represent a communication strategy, as these domains translocate from the ER to other compartments. This is the case of ER-derived quality control vesicles,^264^ which differ from the classical ER-exit COPII vesicles, known to transport, e.g., collagen, lipids and diphenylalanine motif-containing proteins along the secretory pathway.^265^ However, the landscape of such ER-derived vesicles and the extent to which they are involved in redox-related signaling remain poorly explored. We previously identified in vascular smooth muscle cells the presence of actin-encircled cytoplasmic vesicles containing PDIA1, positioned along actin fibers and enriched at membrane ruffles upon cyclic stretch.^4^ In endothelial cells, PDIA1 is detected in vesicles containing GROα chemokine.^266^ In megakaryocytes, PDIs seem to be secreted from cytoplasmic puncta.^267^ In all of these cases, however, it is unclear if these structures are ER-derived or carry PDIA1 from other locations. On the other hand, ER oxidoreductases may be extracellularly secreted within microparticles such as EC vesicles^268^ and exosomes,^269^ which may in part associate with the ER or have their release mediated by ER oxidoreductases themselves. This is in line with evidence for strong thiol redox modulation of biogenesis and secretion of those structures.^268^ For example, PDIA1 secretion via such microparticles in platelets contribute to their aggregation and interaction with coagulation cascade.^270,271^ Cellular calcium derangements may contribute to ER-derived vesicle secretion.^272^ Such a picture aligns with potential roles of PDI family proteins in membrane fusion events including sperm-egg fusion, uptake of viral particles, regulation of calcium fluxes, phospholipid redistribution, and tetraspanin activity. Crosstalk with microparticles can also contribute to the increasingly evident role of PDIs in cancer cell biology and metastasis.^273^ For example, the PDIA1 cargo of extracellularly secreted vesicles is essential for their induced redox-related malignant transformation in bladder cancer cells. Such vesicles are released upon ER stress in donor cells, as a putative way to keep homeostatic levels of PDIA1.^274^ In addition, heme oxygenase-modified bone marrow stem cells, which improve ischemia-reperfusion injury of liver recipient tissue, secrete EC vesicles loaded with ERp72, which in turn promotes reparative macrophage polarization.^275^ In addition to membrane-enclosed microparticles, membrane-less condensates may potentially translocate ER-derived material, such as supermeres, which are RNA-enriched nanoparticles with functions related to neurodegenerative, metabolic, and cardiovascular disease.^276^

### ERDOR via Translocation of ER Oxidoreductases to Extra-ER Locations

Further to the mechanisms discussed so far, the hallmark of the ERDOR is the mobility of ER oxidoreductases such as PDIs away from their primary ER location, a process recently conceptualized as signaling by location.^277^ This is in line with the biochemical scenario of the PDI code discussed above. Again, enrichment of PDIs in small subcellular regions can potentially offset the effects of their low extra-ER levels, acting as a mechanism to focus PDI activity toward specific targets (Table 2). Such locations include cytoplasm, nucleus, endosomes and, in particular, the EC milieu, as follows. There is substantial evidence for an EC (cell surface and secreted) PDI pool, which we came to call “peri-epicellular PDI”.^12^ This quantitatively small pool has several implications. First and most important, such an EC pool is functionally important in pathophysiological events. Moreover, it represents a link between ER and EC redox regulation. For instance, recent work addressing the thiol redox proteomics of endothelial cell surface uncovered a remarkable portfolio of ER oxidoreductases, mostly from the PDI family, together with some structural ER proteins such as reticulon-4, consistent with the ER itself or ER-derived structures showing up at the cell surface.^278^ This agrees with previous reports.^279^ ERp57 has been associated with nongenomic effects of vitamin D3 receptor, which may be of particular importance in the surface of cancer cells.^280^ Together, these data further confirm the important interplay between the ER and EC milieu. ERDOR at locations other than the EC milieu is less well characterized, and functional implications are not as clear. An endosomal location, colocalizing with the Rab5 marker, has been suggested for PDIA1 in the course of VEGFR2 redox signaling and angiogenesis.^241^ Nuclear locations for PDIs have been described, including regulation of thyroid hormone receptor for PDIA1,^281^ a chaperone effect on the estrogen receptor for PDIA1 ^282^ and regulation of gene transcription under stress by nuclear Grp78.^283^ Recently, there has been growing evidence to document a so far elusive cytosolic location for PDI family proteins and ER chaperones. Earlier indirect evidence for such cytosolic location included fractionation and colocalization experiments,^4,238^ as well as physical interactions with cytosolic proteins supporting Nox activation,^284^ actin-related cytoskeletal organization,^285,286^ Drp1-dependent mitochondrial dynamics,^287^ and HIF1α signaling.^288^ The so-called “preemptive quality control” was conceptualized to describe the cytosolic accumulation of prion protein or CRT during ER stress.^66^ Recently, the process of reflux of folded and nonaggregated ER proteins to the cytosol during ER stress was described and termed “ER to Cytosol Signaling”.^289–293^ Refluxed ER proteins may acquire novel function, in particular the PDI family protein AGR2, with its documented inhibitory effect on p53 binding via SGTA cochaperone adaptor,^293^ a mechanism that may drive tumorigenesis.^292,293^ Our^291^ and other^293^ groups showed that silencing the Hsp40 family transmembrane ER chaperone DNAJB12 mitigates PDIA1 ER-to-cytosol translocation during ER stress. Moreover, DNAJB12 overexpression promotes PDIA1 cytosolic translocation^69,291^ and deglycosylation of a Glyco-PDI construct (see below), consistent with DNAJB12 mediating PDIA1 translocation from the ER to the cytosol even in the absence of ER stress.^69^ A similar effect occurs for other PDIs such as ERp57 and ERp72.^294^ This unambiguously indicates that variable amounts of PDI(s) may translocate to the cytosol under some conditions, though the full implications of this event need further exploration.

**Table 2:** An Overview of Molecular Targets Reportedly Regulated by ERDOR

	extracellular or membrane-linked			
	oxidase	reductase	isomerase	redox NS
PDIA1	β1-integrin^317^	β3-integrin^305,344,345^	β3-integrin^305^	thrombospondin-1 ^357^
	α5-integrin^72^	β2-integrin^346^	ADAM-17 ^354^	flippase^358^
	cathepsin G, glutaredoxin-1, GPIb, fibrinogen, thioredoxin^343^ ^,^ ^e^	GP-Ibα^347^	TF^355^	Basigin^d^ ^,^ ^359^
	vWF^305^	β2-glycoprotein I^348^	αMβ2 ^356^	
		αMβ2 ^346^		
		vitronectin^349^		
		factor XI^350^		
		factor V^351^		
		histidine-rich glycoprotein^352^		
		annexin V, heparanase, Erp57, kallikrein 14, kerpin B6, tetranectin, collagen VI^343^ ^,^ ^e^		
		gp120 (HIV)^353^		
ERp57	transglutaminase-2 ^325^	fibronectin^b^ ^,^ ^256^		
		collagens^b^ ^,^ ^256^		
		MASP1–2^e^ ^,^ ^360^		
ERp72		αMβ2-integrin^361^		
ERp46		β3-integrin^305^		
ERp5	β3-integrin^306^			
ERp29	β3-integrin^309^			
TMX1	β3-integrin^310^ (TMEM16F)^362^			
TMX2	SERCA-2^f^ ^,^ ^363^			
TMX4		β3-integrin^364^		
TMX5				HLA class I proteins,
				MHC class I (MICB)^f^ ^,^ ^365^
Trx		β2 glycoprotein I^348,366^ gp120 (HIV)^334^		
Other Locations				
PDIA1	TRPV1 ^324^	granzyme A^369^		estrogen receptor^371^
	Ptp1B^c^ ^,^ ^241^	Drp1 ^287^		Tau^303^
	actin^285^	TEM5′^370^		
	ORF8^a^ ^,^ ^322^			
	p47phox^367^			
	MMP9 ^368^			
ERp57		ficolin-3, collectin 10–11 ^360^		
ERp5		MICA^372^		
ERp44				ORF8^a^ ^,^ ^322^
Ero1α				STIM1 ^231^
Trx		transglutaminase-2 ^325,373^		
		IL-4 ^374,375^		
		CD30 ^376^		
		CD4 ^377^		
		β-defensin^378^		
		TNFR1–2 ^379^		

a From SARSCov2.

b PDIA3 complexed to glycosylated calnexin reduces disulfides in ECM proteins.

c PDIA1 associates with PTP1b sulfenylation.

d Basigin interacts with *Toxoplasma gondii* PDI.

e Substrate trapping interactors.

f Pathogenic mutant of TMX2 mimics an increased oxidative state at MAM.

## Mechanistic Routes of ER Oxidoreductase Translocation: Both Destination and Journey Matter

Signals derived from ER oxidoreductases outside the ER may reflect both their effects at the distinct destinations or during eventual journeys. Thus, understanding of translocation routes is crucial to mechanistically understand how ER oxidoreductases may redox-connect, e.g., the ER-EC compartments and how EC PDIA1, which represents <2% of total cellular PDIA1 pool, e.g., in endothelial cells,^72^ exhibits important physiological effects. Indeed, whether or not PDIs take the classical secretory pathway within this journey can directly affect their physiological effects, redox state,^295^ and possible interactors. The routes of translocation of ER oxidoreductases have been difficult to tackle.^296^ Ero1α and Prdx4 do not have an ER-retrieval KDEL-like sequence, and their location into the ER requires retrieval by association with other KDEL-containing proteins. This is a peculiar effect of ERp44, in a process coordinated with fluxes of zinc dependent on its transporters.^297^ In turn, the most cogent question regarding translocation of PDIs and chaperones (e.g., Grp78 and Grp94) to the cell surface is how they bypass the retrieval function of KDELRs, despite keeping the KDEL sequence to gain the EC space. Interesting models have been proposed in this regard.^66^ While there are reported instances of ER chaperones secreted via Golgi pathway, Golgi-bypass routes have been suggested in several other instances.^72,298^ Overall, there are likely distinct routes for the same protein, although it is unclear whether they balance themselves. Also, these routes are clearly cell-type specific.^72,298^ Work with pharmacological inhibitors and morphofunctional tools in endothelial cells and platelets uncovered possible nonclassical secretion routes for PDIA1 and ERp57.^72,267^ Recently, we adopted the innovative strategy of inserting a glycosylation motif into PDIA1, generating so-called Glyco-PDI, and following the sugar modifications.^69^ These data contribute to unambiguously indicate that PDIA1 is secreted via a yet undefined-possibly novel-nonclassical Golgi-independent secretory pathway in HeLa and endothelial cells. Although secreted PDIA1 bypasses the KDELRs despite keeping the KDEL sequence, deletion of KDELRs drives Glyco-PDI secretion via Golgi, meaning that KDELRs are required for the Golgi-independent PDI secretion.^69^ The KDELRs may normally have a role in trafficking or retention of KDEL-containing proteins, as suggested for CRT^299^ or PDIA1.^300^ It is tempting to propose that cortical ER proteins, by fusing with plasma membrane,^301^ might allow secretion of ER lumen proteins at ER-PMcs. However, our results (DeBessa TC et al., unpublished data) argue against this hypothesis. While such a Golgi-independent route does not involve translocation of Glyco-PDI through the cytosol, our Glyco-PDI tool helped provide clear evidence for PDIA1 cytosolic translocation, as discussed above.^69^ In platelets, actin cytoskeleton disruption abrogates PDIA1 secretion,^267^ while in endothelial cells, cytoskeleton disruption enhances EC PDIA1.^72^ Evidence for a clathrin-dependent recycling uptake route was also described in endothelial cells for PDIA1.^72^ Moreover, there is some evidence that the cell-surface and secreted pools of PDIA1 may be externalized by different mechanisms and not necessarily belong to a single interchangeable pool.^12^ Overall, this picture composes a complex scenario for routes of ER oxidoreductase externalization in cells while being consistent with their tight regulation and physiological importance.

## Molecular Targets and Physiological Implications of ERDOR

A nonexhaustive overview of the molecular targets reportedly regulated by ERDOR is listed in Table 2. It is clear from this scenario that the targets cover diverse protein families with several functions, which are regulated by PDI family members via disulfide reduction, disulfide formation, or mixed disulfide complexation (i.e., thiol oxidation), disulfide isomerization, secretion from cells, and, in a few cases, yet unspecified thiol redox modifications. It is not clear if the predominance of PDIA1 effects over those of other PDIs means its more specific role in ERDOR, just its greater expression levels, or the fact that it has been more well studied. The overall ensuing effects of ERDOR include thrombosis, platelet activation, integrin signaling, virus internalization, pathogen infection, ion flow regulation, membrane lipid organization, immune-inflammatory cell responses, cell adhesion, cell migration, vascular remodeling, mechanoadaptation, cell migration, and ECM remodeling (Table 2). In parallel, additional emerging data have identified intracellular or membrane targets affected by PDIA1 in redox signaling processes related to Nox NADPH oxidases,^165^ actin cytoskeleton,^285^ RhoGTPase activation,^243^ smooth muscle cell differentiation,^302^ angiogenesis signaling,^241^ mitochondrial dynamics,^287^ and prevention of phase separated Tau aggregation,^303^ among others. EC PDI has also other roles in immunomodulation and malignant transformation, reviewed previously.^304^ Here, we comment on a few paradigmatic examples of ERDOR. Additional discussion of these effects can be found in the references and cited reviews.

ERDOR roles in the modulation of integrin family proteins lie at the basis of regulatory effects on thrombosis and platelet activation. In both platelets and endothelial cells, PDI family proteins cooperatively regulate integrins (particularly β3), mainly via reductive cleavage of disulfide bonds and/or an isomerase effect, which cooperatively switch integrins from the resting to the low binding affinity state and from it to the high binding affinity state (maximal activation).^305^ PDIA1, ERp57, ERp72, Erp46, and other PDIs such as TMX4 and Erp18 effects support thrombosis/platelet activation by regulating this mechanism (see refs in Table 2). ERp5 oxidation of a β3-integrin disulfide,^306^ as well as steric hindrance,^307^ inhibits binding of this integrin to fibrinogen, antagonizing platelet function. ER stress emerges as an important regulator of platelet function, and ERp5 deficiency increases platelet ER stress and aggregation.^308^ In addition, Erp29 ^309^ and TMX1 inhibit platelet aggregation, the latter via effects on β3 integrin and TMEM16-mediated phosphatidylserine exposure.^310^ Regulation of GPIb-α and integrin αMβ2 by PDIA1 regulates the platelet–neutrophil interaction and neutrophil migration, respectively. In other cases such as platelet adhesion to hyperglycemic cells, PDIA1 effects associate with secretion of adhesion and integrin-regulating proteins, such as SLC3A2 (CD98) or laminin gamma.^311^ Ero1α reportedly can migrate to the cell surface to support platelet aggregation by forming a local redox pair with PDIA1, to a certain degree reproducing extracellularly the same organization of the ER lumen.^312^ Such Ero1α-PDIA1 interaction associates with remodeling of EC glutathione redox, optimizing the redox potential for platelet activation.^313^ The multiple and complementary effects of ER oxidoreductases in integrins, coagulation factors, and thrombosis-related proteins argue in favor of a redox network integrated by ERDOR extracellularly.^314^

Extensive work from our group unveiled a role for PDIA1 in the regulation of Nox NADPH oxidases in vascular smooth muscle cells, as an essential cofactor for growth factor-activated Nox1 complex and superoxide generation.^3,238^ The mechanisms for this effect include binding to p47phox subunit^315^ and regulation of RhoGTPases and their chaperone RhoGDIα.^316^ PDIA1 effects closely regulate Nox1-related cell migration,^284^ whereas EC PDIA1 regulates mechanoadaptation and force distribution in vascular smooth muscle cells.^317^ Interestingly, while the total PDIA1 pool accounts for the velocity and migration distance, EC PDIA1 fine-tunes migration persistence.^317^ Such effects merge with a key upstream role of PDIA1 on the regulation of vascular smooth muscle phenotype.^302^ In vivo, EC PDIA1 supports expansive vascular remodeling during vascular injury repair^319^ and protects against aortic dissection induced by lysyl oxidase inhibition.^320^ The PDI-Nox axis seems at least to some extent a conserved interaction, also described in the filamentous fungus *Botrytis cinerea*.^321^ Other groups also showed that PDIA1 exerts a crucial role in angiogenesis mediated via VEGFR2.^241^ Also, PDIA1 loss of function promoted mitochondrial fission and endothelial cell senescence, an effect linked to a desulfenylase activity of PDIA1 toward Cys644.^287^

Several studies support roles for PDIs in the internalization or cellular processing of pathogens during infection, including HIV, influenza, or dengue viruses.^3^ SARSCov2 ORF8 protein was documented to form mixed disulfides with several proteins from the ER and secretory pathways. It hijacked in particular PDIA1 and ERp44 to maintain its stability along the secretory pathway, a process potentially favored by the upregulation of Ero1, leading to ER stress.^322^ Redox pathways can also be involved in antigen presentation at the cell surface. Antigen presentation is performed by the ER transmembrane peptide loading complex, which comprises, from its ER side, disulfide bonds between ERp57, or to a lesser degree PDIA1, with tapasin or MHC class I.^323^ Secreted PDIA1 also helps oxidize EC Cys of TRPV1 channels to promote hyperalgesia in dorsal root ganglion sensitive nerves.^324^ In another system, transglutaminase-2 roles in celiac disease depend on its overactivity, which can be redox-regulated by a double oxidoreductase mechanism: intracellular Trx1 reductively activates transglutaminase-2, which is then secreted, while EC ERp57 has a unique oxidative inactivation effect, not shared by PDIA1, ERp72, or QSOX1.^325^ In turn, PDIA1 stimulates the secretion of Wnt3a during neuronal migration.^326^

These selected examples highlight patterns of ERDOR effects on specific targets and together are consistent with regulated network organization for ERDOR.

## Summary, Conclusion, and Perspectives: an Integrated View of the ER Redoxome from Protein Folding to Mediator of Intercellular Redox Communication

The ER redoxome is firmly grounded on its protein folding scenario, canonically enclosed within a membrane-encircled ER compartment. This is indeed appropriate to the extent that only the ER lumen provides the correct redox environment to optimize protein folding and keeps adequate high concentrations of folding factors such as ER oxidoreductases, chaperones, and calcium. In this scenario, the ER produces and productively consumes enormous amounts of oxidants such as H_2_O_2_, while the potential effects of other ROS such as peroxynitrite^327^ remain to be explored. On the other hand, mounting information connects the ER, mainly via the PDI code, to other compartments such the EC milieu. Such connection stems from and extends the general role of ER quality control as a tool to ensure proper communication between intra and EC milieu.^185^ In this context, the ER-EC communication makes sense, considering that both compartments converge regarding their lower reductive redox potentials and higher oxidant concentrations, which differ largely from those of other subcellular compartments (Figure 2). In fact, many models of ER ontogenesis are based on its invagination from the EC milieu.^19^ As a natural sink of entropy and ROS rheostat,^12^ ECM composition and redox regulation play roles in cellular homeodynamics, pathophysiology, and crosstalk with intracellular redox.^328^ That is, PDI externalization may integrate via redox signals ER-dependent proteostasis with intercellular communication and ECM organization. In fact, as previously discussed, a vast majority of EC protein disulfides are formed intracellularly within the ER lumen. At the same time, somewhat paradoxically, the cell surface has a consistent pool of reduced thiols,^329^ which are likely a substrate of oxidoreductases translocating from the ER and/or other locations. It is estimated that ca.25% of these surface thiols are dithiols, i.e., two thiol groups sufficiently close to interact with each other, including the CXXC motifs from Trx superfamily proteins such as PDIs.^330^ Thus, the EC presence of ER oxidoreductases, such as PDIs, may link both compartments via thiol redox chemistry. Moreover, Ero1α can migrate to the cell surface and oxidatively shifts EC glutathione redox potential.^313^ Thus, the ER-based redox pair PDIA1-Ero1α may, to some extent, be replicated extracellularly, potentially contributing to local processing of ECM proteins, as indeed shown for integrins and adhesion proteins. EC/cell surface redox processes also comprise relevant oxidant sources such as Nox NADPH oxidases, which may further reoxidize PDIA1,^165^ particularly in small subcompartments.^331^ This concept is further shaped by our elucidation of Golgi-independent route(s) of PDIA1 externalization, with evidence for pathway(s) of ER-to-EC secretion.^69^ In turn, this further merges with the ER being an evolutionarily shaped upstream regulator of endomembrane redistribution.^332^ Effects of pecPDIA1 improve the reorganization of cytoskeletal mechanoadaptation^286^ and affect numerous molecular targets (Table 2). Cell-surface/pericellular thiol–disulfide exchange or oxidation may also have implications for the cell internalization of proteins or particles. This property is well explored in technology applications; however, the precise mechanisms of thiol-mediated cellular uptake are poorly known.^333^ Possibly, ER oxidoreductases at the cell surface can favor the uptake of EC proteins, microparticles, or condensates through pathways not dissimilar to the well-known PDIA1-mediated uptake of HIV particles via reduction of their GP120 Cys.^333,334^ Some dissimilarities, however, occur between the ER and EC redox landscapes: (1) levels of PDIs and Ero1α, as well as calcium ions, are much higher in the ER vs EC milieu; (2) oxidant sources are diverse, with Nox1/Nox2 more likely at the EC vs Ero1α, Nox4, and mitochondria at the ER milieu.^165^

Similar to other signaling compartments, pecPDI likely redox-shapes specific EC proteins, while contributing to sculpt overall pericellular/cell surface redox landscape.^313^ EC redox remodeling is a natural strategy to regulate cellular immune and inflammatory responses.^41,335^ Reductive remodeling is involved in antigen presentation by dendritic cells to activate T-cells^335^ and other immune processes, promoting optimal cell-surface cytokine activities and TGFβ activation.^335^ How would pecPDIs (pecPDIA1 in particular) sculpt the EC redox landscape beyond their interaction with Ero1α? First, pecPDIs likely redox-interact among themselves and with translocated Trx. Moreover, pecPDIs, alone or via enzymatic oxidant generation such as Noxes, can modulate low-molecular-weight thiol buffers such as cysteine/cystine and reduced/oxidized glutathione.^336^ While such direct action may be somewhat limited given the small amounts of pecPDIA1, its thiol redox modulation of specific targets might amplify such effects, e.g., redox-regulated proteases such as ADAM17, able to cleave EC proteins such as TNFα receptor, amplifying inflammation.^337^ Of note, the way proteins are externalized may affect their EC redox state. Routes utilizing more oxidizing Golgi system tend to secrete proteins with their thiols oxidized, while proteins that need to be secreted reduced (such as rapidly stress-responsive cytokines and danger-signaling mediators) preferentially use Golgi-independent pathway(s).^295^ This may be relevant for PDIA1, which uses Golgi-independent pathway(s) for secretion, unless KDELRs are disabled.^69^ In turn, KDELRs emerge as possible regulators of ER-EC redox crosstalk.^299,300,338^

In summary, the ER redoxome comprises a system fundamentally inserted into its protein folding alma mater while in turn having attributes of an extended integrated signaling network. Such a network redox connects distinct cellular compartments via physical contacts and interacts with other redox subsystems such as mitochondria, peroxisomes, plasma membrane compartments, etc. While the redox pathways underlying protein folding within the ER lumen are relatively well-known, the potential of the extended ER redoxome as a hierarchically regulated integrative cell signaling system has just begun to be explored and promises to have important mechanistic-based physiological implications in intra- and intercellular redox communication.

## Abbreviations list

AQP: aquaporin
ATF: activating transcription factor
CNX: calnexin
CRELD: cysteine-rich epidermal growth factor-like domain
CRT: calreticulin
EC: extracellular
ECM: extracellular matrix
ER: endoplasmic reticulum
ERAD: endoplasmic reticulum-associated degradation
ERDOR: endoplasmic reticulum-dependent outreach redoxome
Ero1: endoplasmic reticulum oxidoreductin 1
ER-PMCs: endoplasmic reticulum plasma membrane contact sites
ER-POCs: endoplasmic reticulum peroxisomes contact sites
FAD: flavin adenine dinucleotide
GPx: glutathione peroxidases
GSH: reduced glutathione
GSSG: oxidized glutathione
IP3R: inositol 1, 4,5-trisphosphate receptor
IRE1: inositol-requiring protein-1
KDEL: endoplasmic reticulum retrieval signal
KDELR: KDEL receptor
MAMs: endoplasmic reticulum mitochondria contact sites
Nox: NADPH oxidases
PDI: protein disulfide isomerase
PERK: kinase RNA-like endoplasmic reticulum kinase
Prdx: peroxiredoxin
QSOX: quiescin sulfhydryl oxidase
ROS: reactive oxygen species
RyR: ryanodine receptor channel
SERCA: sarco/endoplasmic reticulum Ca^2+^ ATPase pumps
SOCE: store-operated Ca^2+^ entry
SOD: superoxide dismutase
STIM: stromal interaction molecule
Trx: thioredoxin
TrxR: thioredoxin reductase
UPR: unfolded protein response
VAP: vesicle-associated membrane protein-associated proteins
VKOR: vitamin K oxidoreductase
